# *In Vitro* Variant Surface Antigen Expression in *Plasmodium falciparum* Parasites from a Semi-Immune Individual Is Not Correlated with *Var* Gene Transcription

**DOI:** 10.1371/journal.pone.0166135

**Published:** 2016-12-01

**Authors:** Ellen Inga Bruske, Sandra Dimonte, Corinna Enderes, Serena Tschan, Matthias Flötenmeyer, Iris Koch, Jürgen Berger, Peter Kremsner, Matthias Frank

**Affiliations:** 1 Institute of Tropical Medicine, University of Tuebingen, Tuebingen, Germany; 2 CERMEL (Centre de Recherche Médicale de Lambaréné), Lambaréné, Gabon; 3 Max Planck Institute for Developmental Biology, Tuebingen, Germany; Institut national de la santé et de la recherche médicale - Institut Cochin, FRANCE

## Abstract

*Plasmodium falciparum* erythrocyte membrane protein 1 (PfEMP1) is considered to be the main variant surface antigen (VSA) of *Plasmodium falciparum* and is mainly localized on electron-dense knobs in the membrane of the infected erythrocyte. Switches in PfEMP1 expression provide the basis for antigenic variation and are thought to be critical for parasite persistence during chronic infections. Recently, strain transcending anti-PfEMP1 immunity has been shown to develop early in life, challenging the role of PfEMP1 in antigenic variation during chronic infections. In this work we investigate how *P*. *falciparum* achieves persistence during a chronic asymptomatic infection. The infected individual (MOA) was parasitemic for 42 days and multilocus *var* gene genotyping showed persistence of the same parasite population throughout the infection. Parasites from the beginning of the infection were adapted to tissue culture and cloned by limiting dilution. Flow cytometry using convalescent serum detected a variable surface recognition signal on isogenic clonal parasites. Quantitative real-time PCR with a field isolate specific *var* gene primer set showed that the surface recognition signal was not correlated with transcription of individual *var* genes. Strain transcending anti-PfEMP1 immunity of the convalescent serum was demonstrated with CD36 selected and PfEMP1 knock-down NF54 clones. In contrast, knock-down of PfEMP1 did not have an effect on the antibody recognition signal in MOA clones. Trypsinisation of the membrane surface proteins abolished the surface recognition signal and immune electron microscopy revealed that antibodies from the convalescent serum bound to membrane areas without knobs and with knobs. Together the data indicate that PfEMP1 is not the main variable surface antigen during a chronic infection and suggest a role for trypsin sensitive non-PfEMP1 VSAs for parasite persistence in chronic infections.

## Introduction

*P*. *falciparum* is responsible for the most severe form of human malaria and is a major cause for morbidity and mortality in sub-Saharan Africa [1]. In endemic areas, semi-immunity against *P*. *falciparum* is associated with the development of antibodies [2,3] against variant surface antigens (VSAs) expressed on infected red blood cells (iRBCs) [4–6].

To date, five multicopy gene families that encode VSAs have been described in *P*. *falciparum*: *stevor* (subtelomeric variable open reading frame) [7], *rif* (repetitive interspersed family) [8], *pfmc-2tm* (*P*. *falciparum* Maurer’s clefts two transmembrane) [9], *surfin* (surface associated interspersed genes) [10] and *var* [11]. However, the antigenic importance of the corresponding VSA protein families is a question of ongoing research. The best investigated VSA is *P*. *falciparum* erythrocyte membrane protein 1 (PfEMP1) [12–14]. PfEMP1 is a variant surface protein that is encoded by the multicopy *var* gene family and mediates cytoadherence of iRBCs to a broad repertoire of host endothelial receptors [14]. In the extracellular part, PfEMP1 possesses a semi-conserved structure consisting of a Duffy-binding like (DBL)-1α domain, a cysteine rich interdomain region (CIDR) located downstream, followed by a variable number of less conserved DBL stretches. Each individual parasite carries approximately 60 *var* genes but only expresses one *var* gene at a time [13–16], thereby ensuring that only one PfEMP1 variant is exposed to the immune system. Switches in *var* gene transcription provide a basis for antigenic variation and immune escape during chronic infections [17] and are tightly controlled on multiple layers. At the level of the individual *var* locus, silencing appears to be mediated by the interaction of the 5’ promoter and the intron promoter as well as histone modifications [18–25]. Epigenetic memory appears to “mark” the active *var* locus [23,26] to ensure its continued expression in the next generation of offspring and it has been shown that individual active promoters are stably transcribed for prolonged periods of time [27]. *in vitro* switching investigations with long term laboratory strains and with parasites obtained from controlled human infections [28–32], provide evidence that *var* gene switching is highly structured and suggest a repeatable hierarchy of *var* gene activation. These observations raise the question of how such a stably inherited transcription pattern is compatible with antigenic variation during natural chronic infections in endemic areas.

Mathematical models [33,34] and serum transfer experiments [35,36] strongly support the seroepidemiological evidence that antibodies against surface antigens are of critical importance in the development of semi-immunity [6,37,38]. Strain-transcending immunity against PfEMP1 has been shown to develop early in life [39,40]. Consistent with this, parasitemia levels in adult residents of endemic areas are often submicroscopic [41]. In contrast, the parasitemia observed during infections of non immune individuals is continuously detected by light microscopy and displays a pattern of consecutive waves with sequential removal and subsequent expansion of parasite populations [33,42]. Hypervariability of the *var* gene family is thought to be necessary for the parasite to escape the human immune response [43,44]. Indeed, frequent recombination events have been documented within the *var* gene family at the individual strain [45–49] and at the population level [50].

In this work we examine the question of how *P*. *falciparum* achieves persistence during a chronic asymptomatic infection by conducting *in vivo* and *in vitro* investigations of a natural infection in an “asymptomatically infected” individual. The *in vivo* data demonstrated persistence of the same parasite strain throughout the infection. Fluorescent-activated cell sorting (FACS) analysis of cultured adapted parasites with convalescent sera identified a clonally variant surface recognition signal that was not associated with *var* gene transcription. This signal could be abolished by trypsinisation and immune electron microscopy revealed that antibody binding was in membrane areas with and without knobs. Together the data suggest that the surface signal of infected red blood cells from a chronic infection is not exclusively composed of PfEMP1 and support a role for trypsin sensitive non-PfEMP1 VSAs in parasite persistence during chronic infections.

## Results

### A stable parasite population survives for an extended time period during an asymptomatic *P*. *falciparum* infection

**Fig 1:**
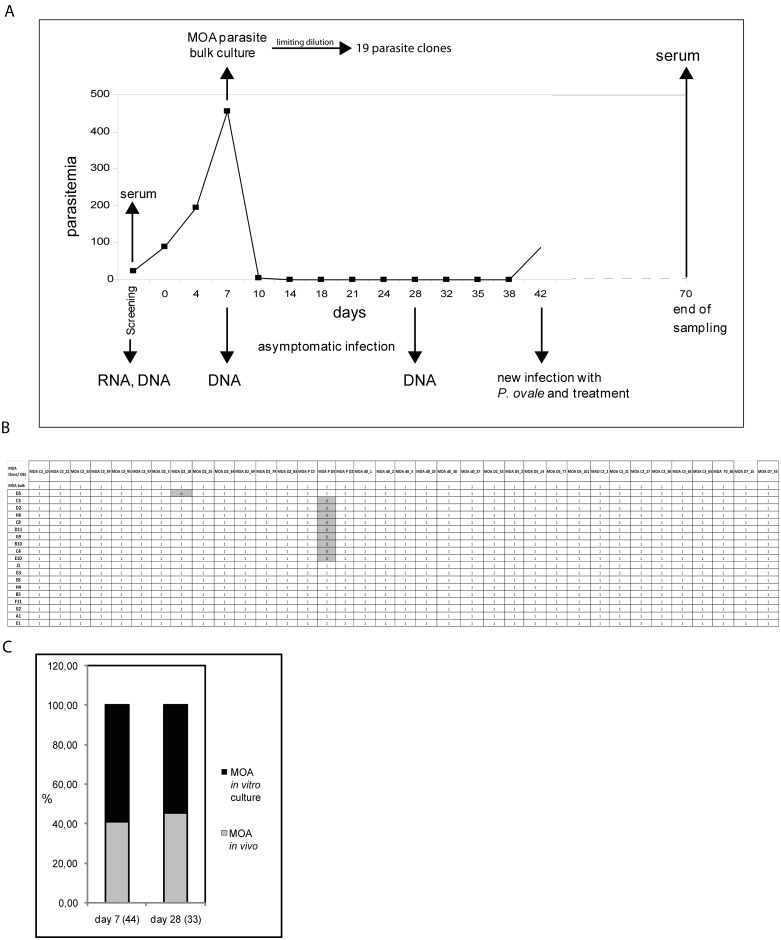
*In vitro* and *in vivo* analysis of a chronic *P*. *falciparum* infection in a semi-immune individual. (A) Parasitemia curve of the asymptomatic chronic *P*. *falciparum* infection of an adult semi-immune Gabonese individual over 70 days and timeline of the *in vivo* investigation. The patient was asymptomatic from day 0 to day 42, at which point a new infection with *P*. *ovale* was diagnosed and treated according to local guidelines. DNA, RNA, cryopreserved parasites and sera employed in this analysis are indicated by arrows. Blood parasitemia is quantified on the y-axis. *P*. *falciparum* parasites were detected from day 0 to day 10 by light microscopy. From day 7 to day 38 sub microscopic parasitemia was documented by PCR twice per week. For *in vitro* analysis parasites from day 7 were brought into tissue culture (MOA bulk) and 19 *in vitro* clones were generated by limiting dilution in two independent cloning experiments. (B) Matrix of PCR results with 36 DBL specific primers on DNA of *in vitro* MOA bulk culture and 19 MOA clones. 1 indicates amplification of the target sequence, 0 indicates absence of a reaction product. All 36 DBLs were amplified from MOA bulk and the clones MOA J1, G3, E8, H4, B5, F11, G2, A1 and E1. In the remaining clones 35 DBLs were successfully amplified. In the clone MOA D5 the DBL MOA D2_18 could not be amplified. In the clones MOA C3, D2, H6, C8, D11, G9, B10, C4, E10 the DBL MOA P D5 could not be amplified (grey). (C) Results of DBL shotgun cloning of DNA from patient blood of day 7 and day 28. Numbers in brackets shown below the x-axis refer to the total number of *var* DBL sequences identified on these days. Sequences that were identified exclusively in DNA from patient blood are designated as "*in vivo*" MOA (grey). The proportion of MOA “*in vitro*” DBLs is represented by the black part of the bars. MOA *in vitro var* DBLs were detected at the same proportion (59% (26 of 44)) on day 7 and day 28 (55% (18 of 33)).

We investigated the dynamics of a *P*. *falciparum* infection in an asymptomatically infected Gabonese individual (MOA) for a period of 42 days (Fig 1A). Parasitemia increased from 90 parasites/μl on day 0 to a maximum of ~ 450 parasites/μl on day 7. Between day 7 and day 14, blood parasitemia dropped in the absence of treatment and remained submicroscopic until day 42 of the infection. On day 42 the patient became symptomatic with a *P*. *ovale* infection and was treated according to local guidelines. Despite negative thick blood smears from day 14 to 42, we detected a continuous submicroscopic parasitemia by *msp2* FC allele specific PCR. To determine if the obtained *msp2* signals indeed represented the same parasite population, we sequenced the PCR products in MOA blood samples. The PCR products from day 7, 14, 21, 28 and 42 showed identical *msp2* FC allele sequences (accession numbers KC887547-KC887556). These data suggested that the parasitemia consisted of a stable parasite population. We have previously shown that parasites with identical *msp2* sequences can still represent different strains with different *var* gene repertoires [47]. To determine the presence of individual parasites throughout the infection we therefore characterized the *in vivo* and *in vitro var* gene repertoire. To this end, a MOA parasite isolate of day 7 (MOA bulk) was transferred into *in vitro* culture and 19 clones were generated in 2 independent limiting dilution experiments. The first limiting dilution experiment generated the 3 clones MOA D2, C3 and D5 and has been previously reported [30]. The second limiting dilution experiment generated 16 new MOA clones: H4, G3, E8, B5, J1, E10, C4, C8, G9, E1, B10, D11, F11, H6, A1 and G2.

We characterized the DBL alpha repertoire of the MOA clones with universal DBL primers [30]. This revealed 36 individual DBLα sequences (Fig 1B) (accession numbers KC887669-KC887743). All DBLα sequences were confirmed by PCR with DBL specific primers followed by Sanger sequencing. This MOA DBL primer set was used to compare the *var* gene repertoire of the MOA bulk culture and the 19 MOA clones. As expected, PCR on genomic DNA (gDNA) of the orginal MOA bulk culture resulted in PCR products for each of the 36 DBLα sequences. 35 primers produced PCR fragments on all 19 MOA clones (Fig 1B). In one clone (MOA D5), the MOA D2_18 DBL could not be amplified. In 9 clones (MOA C3, D2, H6, C8, D11, G9, B10, C4, E10) the MOA PD5 DBL could not be amplified and in the 9 remaining clones (MOA J1, G3, E8, H4, B5, F11, G2, A1, E1), all 36 primer pairs resulted in PCR products. Together, the data suggested that the clones were isogenic but recombination events had occurred in individual parasite clones.

To identify the relative proportion of *in vitro* MOA DBLs throughout the *in vivo* infection, we determined the *var* gene repertoire of DNA obtained on day 0, 7 and 28 (accession numbers KC887557-KC887668) of the infection by the same DBL cloning approach. Sequence comparison revealed that of the 44 *var* gene sequences identified on day 7, 26 (59%) corresponded to *var* genes identified within the repertoire of the MOA *in vitro* clones. Of the 33 *var* sequences that were identified on day 28, 18 (55%) corresponded to MOA clone *var* genes. The relative proportion of MOA *in vitro* DBL sequences thus remained stable over time (Fig 1C). The remainders of the sequences on day 7 or day 28 were not identified in the *in vitro* DBL MOA cloning experiment thus indicating the presence of clones, which were not identified in the *in vitro* limiting dilution experiment. We next wanted to investigate, if *var* sequences, which had not been cloned on all days, were present throughout the course of the infection. We designed 11 primer pairs for DBL sequences (S1 Table). All primer pairs successfully amplified PCR products from DNA of all three days, which were confirmed by sequencing. DBL sequence analysis revealed a few chimeric sequences that were only identified on individual days of the infection. However, PCR with chimera specific primers could not verify the existence of the sequences (data not shown), indicating that they were generated during the PCR with conserved DBL primers, a problem that has been reported previously [46].

DBL cDNA cloning on day 0 of the infection in MOA blood samples revealed that in marked contrast to the sequence diversity detected in the DNA cloning experiments, only 6 different *var* transcripts were detected. All of the 6 *var* transcripts were identified within the *var* gene repertoire of MOA *in vitro* clones and are shown in Table 1. Furthermore, *in vivo* transcription was biased towards one specific DBL: d0_37. Subsequently, the transcription of these 6 *var* genes was assessed by quantitative Real-Time PCR with gene specific primers in the culture adapted MOA bulk strain at 30, 90 and 150 generations of *in vitro* growth. This revealed stable transcription of all 6 *var* loci at all time points, suggesting relatively low switch rates of these 6 *in vivo* transcripts during *in vitro* growth (S1 Fig).

**Table 1:** MOA *in vivo* DBL transcripts and corresponding *in vivo* DNA DBLs.

	MOA *in vivo var* transcripts
**1**	d0_37 / Day7_18
**2**	d0_30 / Day7_CL24
**3**	d0_2 / Day7_92 / Day28_74
**4**	d0_3 / Day7_61
**5**	d0_1/ Day7_35 / Day28_2
**6**	d0_23

The 6 *in vivo* DBL-transcripts are denoted d0_37, d0_30, d0_2, d0_1 and d0_23. The corresponding *in vivo* MOA DNA DBLs from days 7 and 28 are also indicated. All *in vivo* transcripts were present in the 19 MOA clones (Fig 1B). Among 6 identified individual *var* transcripts, d0_37 was most abundant in two independent cloning experiments. Transcription of the cDNA *var* DBLs was confirmed by Quantitative Real-Time (qRT) PCR with gene specific primers (data not shown).

### Convalescent serum of the MOA individual displays a variable surface recognition signal on different MOA clones

**Fig 2:**
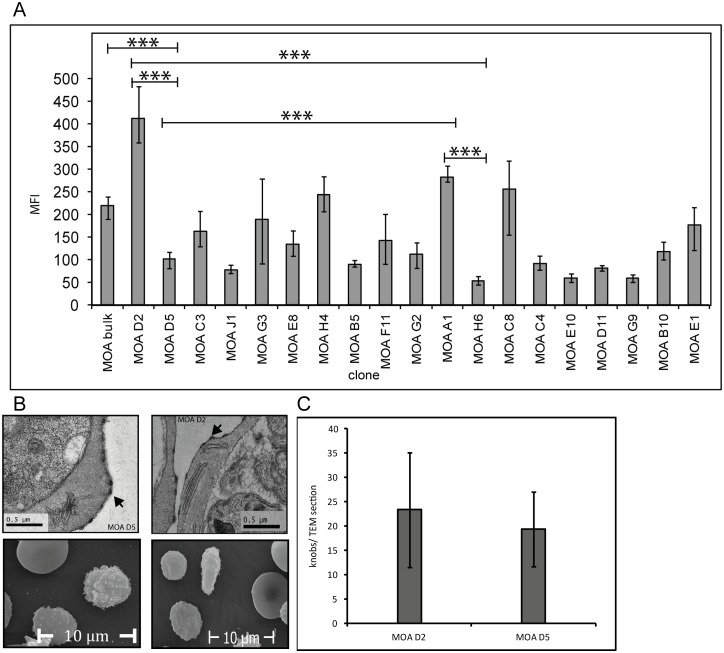
Flow cytometry signals with day 70 serum on MOA bulk and MOA clones are variable. (A) Mean fluorescence intensities (MFI) obtained with MOA serum of day 70 followed by staining with a secondary antibody attached to FITC shows variable surface recognition signals ranging from low (< 80 MFI) to medium (81–160 MFI) and high (> 160 MFI) in MOA bulk and in the MOA clones. Error bars reflect the mean error of at least three independent experiments (Student´s t-test: MOA bulk vs. D5: p = 0.008, A1 vs. H6: p = 0.0003, D2 vs. D5: p = 0.0001, D2 vs. H6: p = 0.008, D5 vs. A1: p = 0.0002). (B) and (C) show transmission and scanning electron microscopy graphs of two representative clones with a highly significant difference in surface recognition signal (MOA D2 and MOA D5) and the presence of knobs (arrows) in equal numbers. Knobs were quantified per TEM cut of each clone.

To test whether a part of the parasite population was able to avoid the host´s immune system, we investigated the humoral immune response against surface antigens of the MOA bulk strain and the 19 MOA clones with convalescent serum obtained on day 70 of the investigation. As expected, surface recognition signals of the MOA clones in flow cytometry were variable -ranging from a mean fluorescence (MFI) of 54 to 377- and significantly different from each other (Fig 2). The signal of MOA bulk (MFI of 174) reflected the mean value of all clones contained in this culture.

PfEMP1 presentation in the membrane of the infected erythrocyte has been shown to differ in knob positive and knob negative parasite lines [51,52]. To ensure that the difference in FACS signal was not due to a loss of knobs in some strains, we performed raster- and scanning electron microscopy on two clones with a highly significant difference in FACS signal: MOA D2 (MFI = 377) and MOA D5 (MFI = 93). This demonstrated that both clones carried knobs. Observer blinded knob quantification showed no quantitative knob difference in the two clones. Furthermore, the *kahrp* gene could successfully be amplified from DNA of all clones (data not shown).

### *var* gene transcription and surface reactivity do not correlate with each other

To assess if the differences in surface reactivity correlated with transcription of individual *var* loci, we investigated *var* gene transcription by DBL specific Real-Time PCR in all 19 clones and the MOA bulk culture. The transcription signal was quantified as the relative copy number (RCN) of the housekeeping gene arginyl-tRNA synthetase (PFL0900 c).

The 3 MOA clones D2, C3 and D5 that were generated in the first limiting dilution experiment were analyzed after > 65 generation of continued growth since limiting dilution. The 16 MOA clones generated during the second limiting dilution experiment were analyzed after approximately 35 generations of continued growth since limiting dilution.

**Fig 3:**
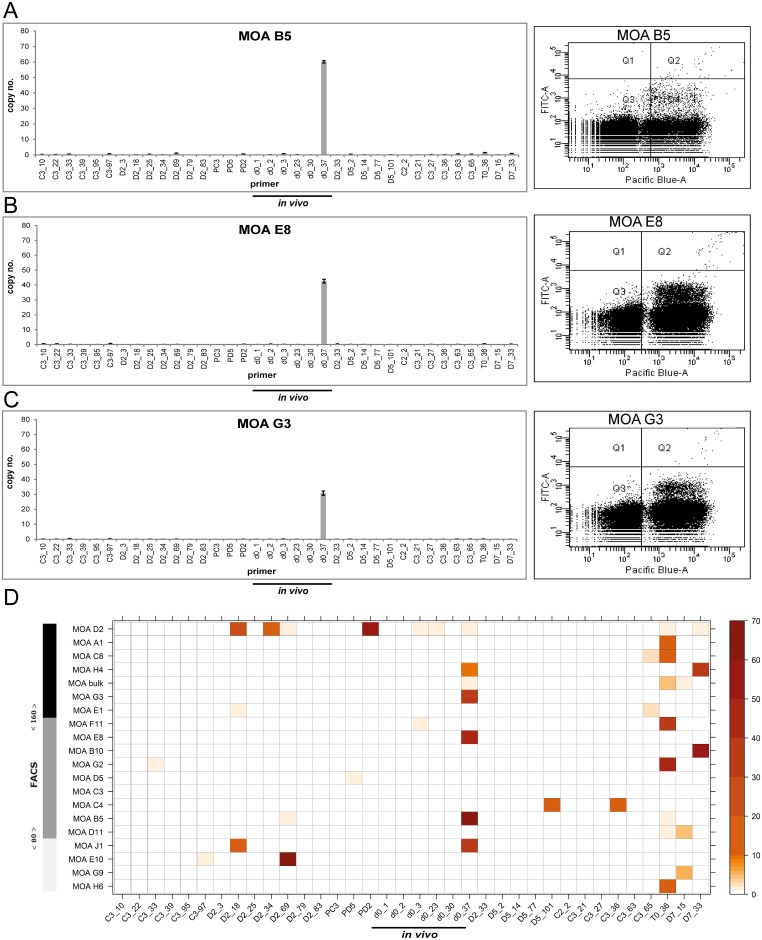
No correlation of *var* gene transcription and flow cytometry signals in clonal parasite populations. (A) Left panel: *var* gene transcription profile of the clone B5 transcribing the MOA *in vivo* transcript d0_37 with the highest transcription signal. The transcription signal is quantified as relative copy number (RCN) of the housekeeping gene arginyl-tRNA synthetase (PFL0900 c) (n = 3, standard errors are given) on the y-axis. The 36 primer pairs are depicted on the x-axis. The 6 primer pairs quantifying *in vivo* transcripts are underlined. Right panel: The corresponding dot plot after incubation with MOA serum of day 70. DNA signals by staining with Hoechst-33342 are depicted on the x-axis ("Pacific Blue-A"), the antibody recognition signal is depicted at the y-axis ("FITC-A"). Uninfected red blood cells are shown in area Q3, infected erythrocytes with both strong (upper cloud) and weak (lower cloud) signals accumulate in Q4. (B) and (C) *var* gene transcription profiles of clones MOA E8 and MOA G3 transcribing d0_37 at lower transcription signals but exhibiting higher surface signals than clone B5. (D) Heat map correlating *var* gene transcription and flow cytometry signal in 19 MOA clones and the MOA bulk culture. The FACS signal is quantified by the left bar with a colour code ranging from black (high: MFI > 160) to dark grey (medium: MFI 81–159) to light grey (weak: MFI < 80). All MOA clones and MOA bulk are sorted according to their corresponding flow cytometry signal and depicted on the y-axis. The MOA specific primer set for 36 *var* loci is listed on the x-axis. *var* gene transcription is colour coded as indicated by the bar on the right. *var* genes marked in red are those with the highest transcription signal and *var* genes in white are those with very low (<3%) or no transcription. Note that clones transcribing d0_37 and T0_36 are evenly distributed from high to low flow cytometry signals.

**Fig 4:**
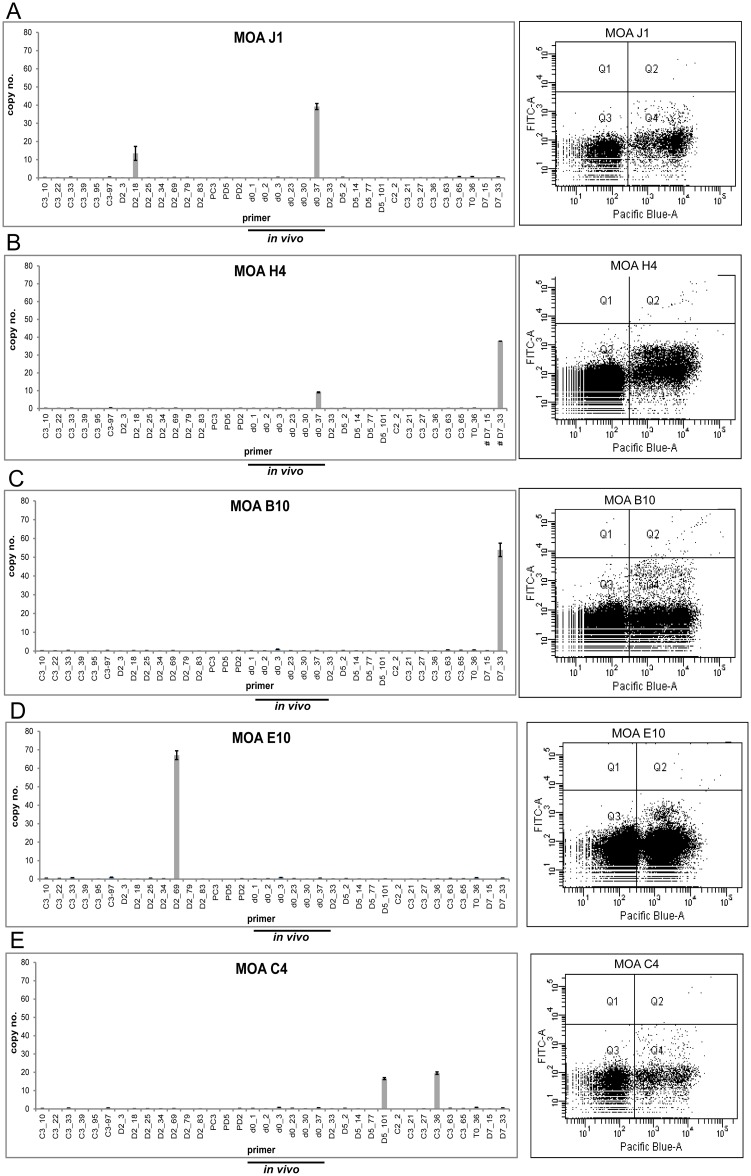
No correlation of FACS signal and switching or transcription strength at 35 generations after cloning. (A) Clone J 1 transcribes the *in vivo* transcript d0_37 and additionally DBL D2_18 but exhibits a low surface signal (MFI of 71). (B) and (C) Transcription of d0_37 and DBL D7_33 in Clone H4 is associated with a high surface signal (MFI of 223.67), but exclusive transcription of DBL D7_33 in clone B10 has a medium surface signal (MFI 109). (D) The exclusive transcription of D2_69 in clone E10 (at the highest individual transcription signal of all clones) is associated with low surface signal (MFI of 55.33). (E) Clone C4 Transcription of D5_101 and C3_36 at close to identical copy numbers. The clone has a medium MFI of 84.

In 5 of the 16 MOA clones generated in the second limiting dilution experiment the *in vivo* transcript d0_37 was the dominant *var* gene transcript (H4, G3, E8, B5, J1) but the height of the transcription signal was not correlated with surface reactivity (Fig 3A–3C). In order to investigate the correlation of surface reactivity and *var* gene transcription in the entire population, a heat map correlating the *var* transcription signal and the individual FACS signals of all MOA *in vitro* cultures was generated (Fig 3D). This showed that the 5 clones transcribing d0_37 showed high, mean and low FACS signals. Furthermore 5 clones (G2, C8, A1 H6 F11) transcribing T0_36 as the dominant transcript exhibited FACS signals from high (A1) to low (H6) (S2 Fig). Analysis of individual clone transcription profiles showed that neither transcription of two dominant *var* loci (indicating a transcription switch in part of the population) nor individual high *var* DBL transcription signals were correlated with the height of the surface signal (Fig 4 and S3 Fig).

We next compared the transcription signal of the dominant *var* loci in all 19 clones and the MOA bulk culture (S3 Table). The strength of the dominant *var* locus in 16 clones (35 generations of growth since limiting dilution) ranged from 67 RCN (DBL_D2_69, Clone E10) to 3 RCN (DBL C3_65, Clone E1). The strength of the individual *var* signals in the clones transcribing d0_37 as the dominant *var* locus ranged from 60–30 RCN and from 42–19 RCN for the clones transcribing T0_36,clearly indicating that these were the dominant transcripts.

**Fig 5:**
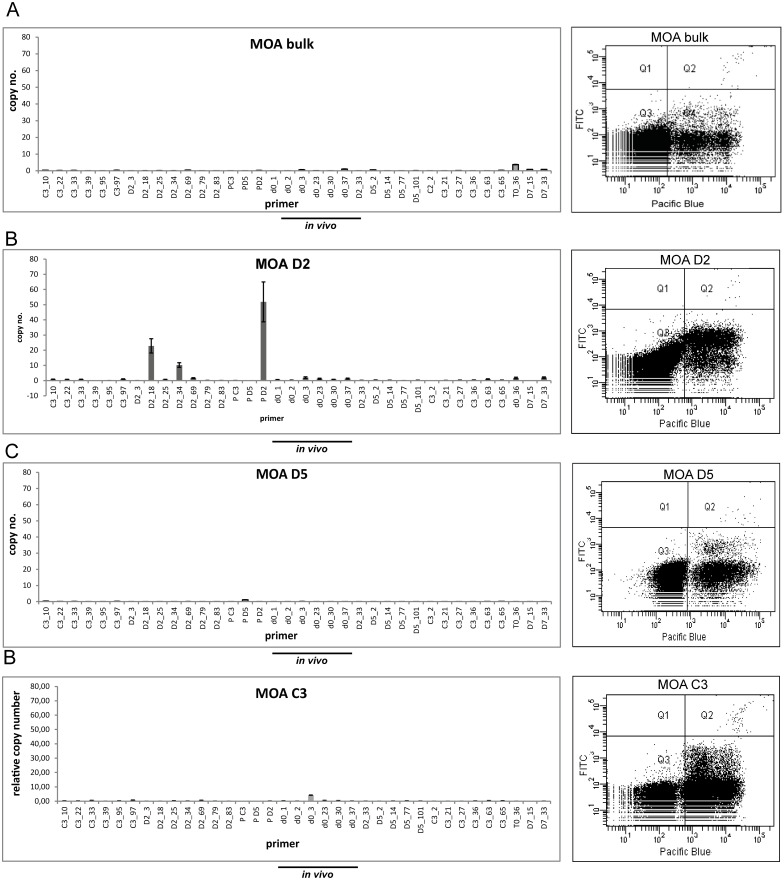
No correlation of FACS and *var* signals in MOA bulk and clones at >65 generations. (A) The MOA bulk culture exhibits low *var* gene transcription signals, but a high MFI (201). (B) Clone D2 exhibits the highest MFI (489) in the entire population and high *var* gene transcription signals for transcripts P_D2 and D2_18. (C) and (D) Clones D5 and C3 display low *var* gene transcription signals but show MFIs in the medium range (MFI of 63.67 and 139.67 respectively).

As expected the MOA bulk culture and the clones C3 and D5 (> 65 generations of growth since limiting dilution) showed lower maximum individual *var* gene signals (3, 1 and 4 RCN). Despite lower transcription signals the surface signal of the MOA bulk culture and the clones C3 and D5 was high and medium (Fig 5A, 5C and 5D). However clone D2 (> 65 generations of growth since limiting dilution) displayed persistently high individual *var* gene signals of the DBLs PD2 and D2_18 and exhibited the highest surface recognition signal of the entire population (Fig 5B). Overall, there was no correlation between the height of the surface recognition signal and *var* gene transcription in the entire population.

### Convalescent serum has strain-transcending anti-PfEMP1 immunity against laboratory strains

**Fig 6:**
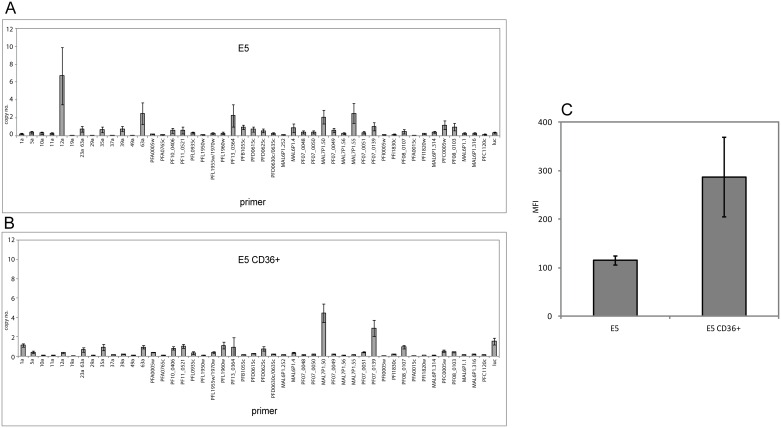
FACS signal with MOA day 70 serum of the laboratory strain E5 increases after CD36 selection. (A) *var* gene transcription profile of the laboratory clone E5 prior to CD36 receptor binding selection. Transcription was quantified with an E5-specific primer set (x-axis) (n = 3, standard errors are given). (B) E5 *var* gene transcription after 3 consecutive rounds of selection for binding to the CD36+ receptor. (C) Surface antigen recognition measured by flow cytometry with serum of MOA d70 shows a stronger signal for the CD36-selected E5 lab strain (standard errors given, n = 3).

To investigate if the convalescent MOA serum had cross-reactive anti-PfEMP1 components, we selected E5 and NF54-C2, two sibling parasites of the original NF54 laboratory strain, in three consecutive CD36 receptor panning assays [31,47]. Unselected E5 had a comparatively low surface reactivity in flow cytometry using MOA serum of day 70 with a mean fluorescence of 114. CD 36 receptor binding selection resulted in an increased surface recognition signal of E5 CD36+ to MFI = 287 compared to wild type E5, when exposed to heterologous serum of the MOA individual from day 70 (Fig 6). The same increase in MFI was observed for NF54–C2 after CD36 selection (S4 Fig). *var* gene profiling in the E5 strain was done using an expanded primer set by designing primers based on previously published DBL sequences [47] and two newly identified DBLs (S3 Table) (accession number KC887546). For NF54-C2 transcriptional profiling we utilized a gene specific primer set [22,30]. CD36 binding selection changed the transcription profile in E5 and NF54–C2, indicating that the phenotype was likely mediated by PfEMP1 (Fig 6 and S4 Fig). In contrast to the laboratory strains, binding to the CD36 receptor in MOA D2, D5 and C3 field isolates was very weak and consecutive rounds of panning did not result in a significant increase in CD36 binding capacity (Table 2). Together these data show that laboratory strain CD36-binding PfEMP1 are recognized by the MOA serum antibodies.

**Table 2:** CD36 receptor binding can be increased in laboratory strains but not in culture adapted field isolates.

strain	Binding/ 50 C32 nuclei before selection	Binding/ 50 C32 nuclei after selection
NF54	11	164
E5	12	232
MOA D2	2	11
MOA D5	15	1
MOA C3	0,7	15

Binding of the trophozoites to the CD36 receptor at the surface of C32 melanoma cells was quantified after several consecutive rounds of panning. The numbers depict the amount of bound *Plasmodium* trophozoites per 50 C32 cell nuclei. Adhesion of the laboratory strains NF54 and E5 was increased, but binding of MOA D2, D5 and C3 was not influenced.

### The surface signal of the MOA D2 clone is PfEMP1 independent

**Fig 7:**
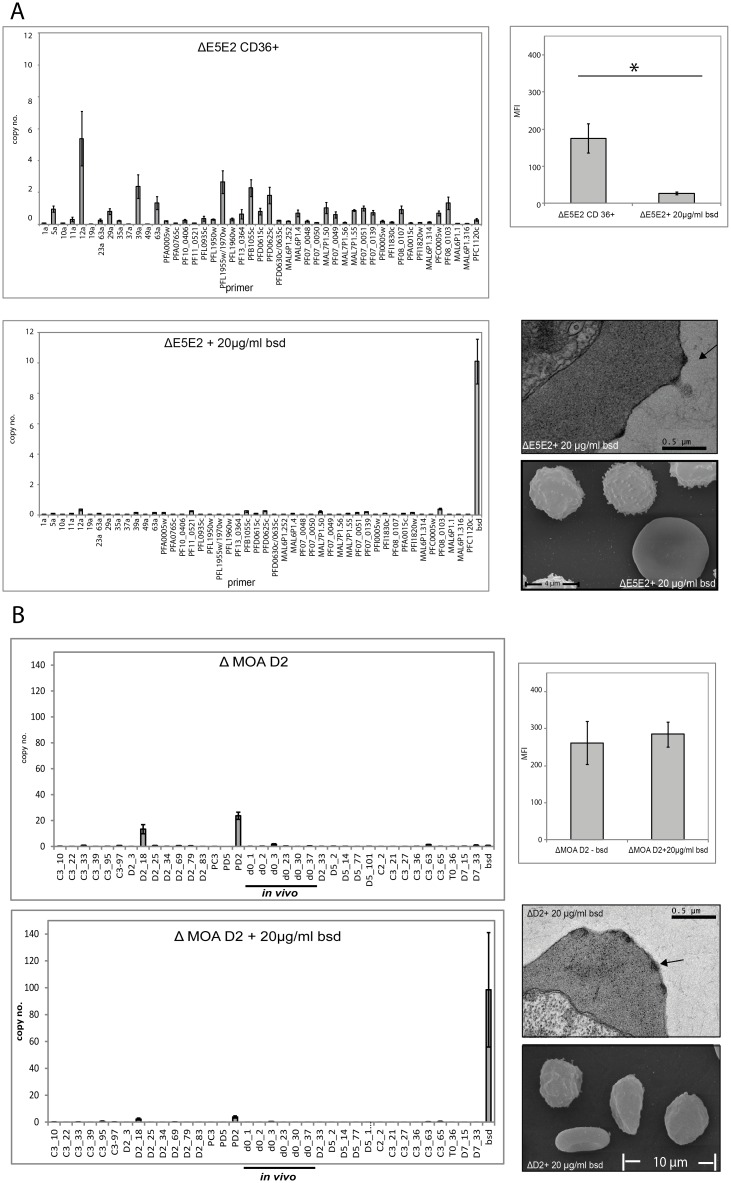
*var* knock-down removes the FACS signal in NF 54 E5 but not in MOA D2. (A) *var* gene transcription profiles of the transgenic ΔE5E2 strain. The upper panel shows the *var* gene transcription profile of the ΔE5E2 cell line after CD36 receptor binding selection without blasticidin pressure. The lower panel depicts the same cell line after *var* gene knock-down. The corresponding flow cytometry signals (serum of day 70) are shown in the upper bar graph on the right. The mean fluorescence (MFI) is significantly reduced in the knock-down cell line (standard errors are given, p = 0.02, n = 4). The transmission electron microscopy (TEM) picture and the scanning electron microscopy picture (SEM) show that the E5 *var* knock-down cell line has intact knobs. (B) *var* gene transcription profile of transgenic MOA ΔD2 cell line without (upper graph) and with (lower graph) blasticidin pressure. Flow cytometry with serum of day 70 does not reveal any difference in their surface recognition signals of the two cell lines (standard errors are given, n = 3). The TEM and SEM pictures of MOA ΔD2 also clearly identify knobs in the erythrocyte surface membrane.

To specifically determine the target of the MOA serum on the infected erythrocyte in the different parasite lines, the transgenic *var* knock-down parasites ΔE5E2, ΔMOA D2 and ΔNF54-C2 were generated. The MOA D2 line was chosen for this experiment because it was the clone with the highest surface recognition signal and showed constant transcription of the DBLs PD2 and D2_18 despite prolonged *in vitro* growth (Fig 5). In these parasites, application of blasticidin drug pressure leads to the transcriptional shutdown of the *var* gene family [22,53]. The transgenic lines ΔE5E2 and ΔNF54-C2 were selected for CD36 binding in the absence of blasticidin drug pressure. As expected, this generated strong surface reactivity with MOA d70-serum (Fig 7 and S5 Fig). Application of blasticidin resulted in transcriptional knock-down of the *var* gene family and a strong reduction of surface reactivity in ΔE5E2 (Student´s t-test p = 0.02) and ΔNF54-C2 (Fig 7 and S5 Fig). Furthermore, CD36 receptor binding selection of ΔE5E2s and ΔNF54-C2 was not possible under blasticidin pressure (S5 Fig) despite the presence of knobs in the membrane of the infected erythrocytes (Fig 7A).

Transcriptional profiling of the transgenic line ΔMOA D2 (grown without blasticidin pressure) displayed a strong surface signal and strong transcription of endogenous *var* DBLs PD2 and D2_18. After growth under blasticidin pressure, the transcription of PD2 and D2_18 was reduced to background levels and the *bsd* locus was the dominantly transcribed *var* gene/promoter. Despite this, both lines exhibited an identical surface signal in flow cytometry with orthologous, convalescent serum from day 70 (Fig 7B). The data show that in contrast to the transgenic parasite line ΔE5E2 and ΔNF54-C2, the antibody recognition signal of ΔMOA D2 is not affected by PfEMP1 knock-down.

### Surface antigens of NF54 E5 and MOA D2 are trypsin sensitive and located in membrane areas with and without knobs

**Fig 8:**
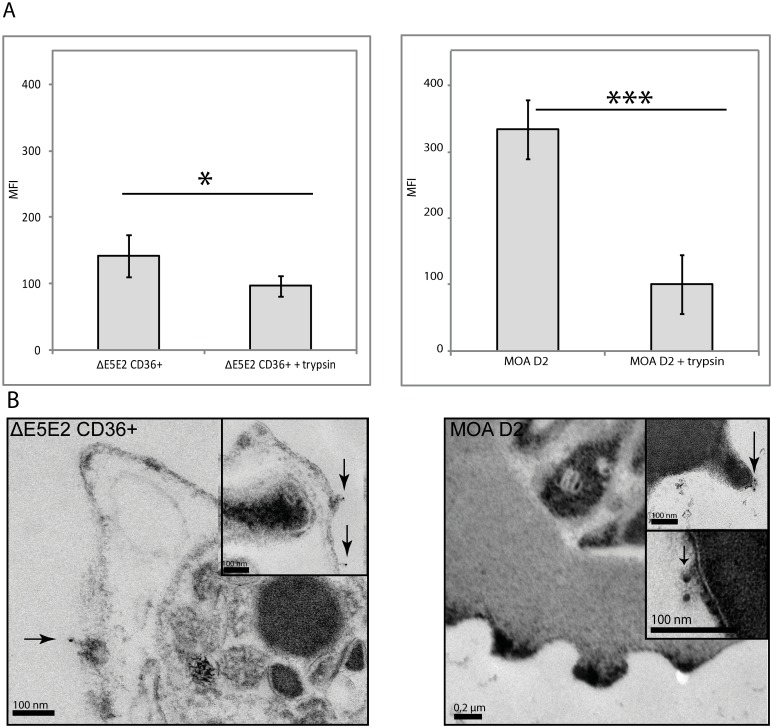
The MOA D2 surface recognition signal is trypsin sensitive. (A) Surface recognition signal with day 70 sera before and after trypsinisation of CD36-selected ΔE5E2 (left panel) and of MOA D2 (right panel). Both ΔE5E2 and MOA D2 were incubated with MOA serum of day 70 and labelled with a secondary FITC antibody for detection in flow cytometry. Trypsinisation resulted in a significant decrease of the antibody recognition signal (standard errors are given, p = 0.05 for ΔE5E2 (n = 4) and p = 0.003 (n = 3) for D2) in both cell lines. (B) Immuno-TEM after MOA day 70 serum labelling on CD36 selected ΔE5E2 parasites and MOA D2 parasites. A secondary, 12 nm gold-labelled anti-human IgG antibody was added. In ΔE5E2 and MOA D2, gold particles (marked with arrows) could be detected on membrane areas with knobs as well as on the membrane areas without knobs.

To further characterize the targets of the MOA antibodies on infected erythrocytes, ΔE5E2 CD36+ and MOA D2 were trypsinised. Trypsinisation and thereby shearing of the proteins on the erythrocyte surface of ΔE5E2 CD36+, which initially had a strong flow cytometry signal with MOA d70 serum, resulted in a reduction of the surface signal (one-tailed paired t-test p = 0.05) (Fig 8A). Trypsinisation of MOA D2 wild type resulted in highly significant signal reduction (p = 0.003). MOA antibodies thus clearly detected trypsin sensitive surface antigens in both strains. To test if the surface signal of MOA d70 serum was associated with knobs, electron microscopy and staining with gold-labelled anti-human IgG antibodies after incubation with serum of day 70 was performed. In ΔE5E2 CD36+ and in MOA D2 gold particles were identified on knobs and on membrane areas without knobs (Fig 8B). This demonstrates that the MOA serum targets knob-independent and knob-associated surface antigens.

## Discussion

In semi-immune individuals living in high transmission areas, parasites are not eradicated completely from the host, but do survive at submicroscopic levels in the bloodstream. Consequently, *P*. *falciparum* faces increasing immunologic pressure during chronic infections [4,54,55]. Here we try to address the determinants of *P*. *falciparum* persistence during a chronic asymptomatic infection of a semi-immune Gabonese individual. We show that the same individual parasite clones survive for at least four weeks in the host, although at drastically reduced parasitemia levels.

Historic serum transfer experiments have shown that transfusion of sera from a semi-immune adult will clear the infection of a child with malaria or a non-immune adult individual with malaria [2,3,36]. The decrease of parasitemia without any therapeutic intervention to submicroscopic levels we observed in our patient is clearly consistent with these results. This raised the question how the parasites were able to escape the immune response, rendering long-term persistence possible. To characterize the surface antigens of the MOA isolates, convalescent MOA serum (day 70) was employed. This ensured enough time for the generation of anti-parasitic antibodies against the day 7 parasites investigated in this work. MOA serum FACS analysis with CD36 selected NF54 clones and NF54 PfEMP1 knock-down parasites clearly showed strong strain transcending anti-PfEMP1 reactivity, confirming that PfEMP1 is a major VSA target of the humoral immune response. FACS analysis of the isogenic MOA parasite clones showed a variable surface recognition signal, clearly supporting the role of VSAs in prolonged parasite persistence. Surprisingly, the surface recognition signal did not correlate with transcription of specific *var* genes.

Assessment of *var* gene transcription in NF54 clones is facilitated by the availability of the entire genome sequence [13]. In contrast, assessment of *var* gene transcription in a field isolate is complicated by the seemingly endless genetic diversity of this gene family [43]. To enable *var* gene specific transcription profiling in the MOA parasite, we developed a MOA specific primer set covering 36 of the presumed 60 MOA *var* genes. It was therefore possible that the observed dissociation of *var* gene transcription and surface signal in the MOA clones might have been simply a consequence of the inability of the primer set to detect specific *var* gene transcripts. Several aspects make this highly unlikely. *In vivo var* gene transcription analysis by cDNA DBL cloning identified d0_37 as the dominant *var* transcript. After tissue culture adaptation of the MOA bulk parasite, qRT PCR analysis with a newly designed MOA strain specific primer set identified d0_37, T0_36 and D7_15 as the dominant transcripts. Thus d0_37 was detected as the dominant transcript *in vivo* and *in vitro* by two different methods. After limiting dilution five MOA clones transcribed d0_37, five transcribed T0_36 and two transcribed D7_15 as the dominant transcripts. All other dominant transcripts were only detected once in the population of 19 clones. The transcript distribution among the MOA clones therefore clearly reflected the transcription pattern of the MOA bulk culture. Furthermore, the maximum transcription signal of the clones transcribing d0_37 and T0_36 was very high (60 and 42 RCN respectively) strongly suggesting that these were indeed the dominant transcripts. To verify the qRT PCR results of the MOA clones, a second method to identify the domimant *var* transcript (cDNA PCR with conserved DBL primers followed by Sanger sequencing) was performed. This confirmed the identity of the qRT PCR transcript in 15 of 16 clones (the exception being the clone E1).

The surface signal of iRBCs with sera from semi-immune individuals has recently been employed by several investigations as a marker for PfEMP1 expression in NF54 parasites. The surface reactivity of sera from Kenyan children against CD 36 selected 3D7 wild type parasites increased with age and was consistently higher than against 3D7 *var* knock down parasites [40]. Similarly, transgenic parasites devoid of the SBP1, a transport protein involved in the trafficking of PfEMP1 to the surface, were shown to display a decrease in surface reactivity with sera from children and adults from Kenya, Malawi and Papua New Guinea [56]. Consistent with this, CD 36 receptor binding selection of the NF54 clones in this work was associated with transcriptional switches among the *var* gene family and this resulted in an increase in surface signal with the MOA serum.

In addition recent data from controlled human infections show that PfEMP1 expression is changed by intrahost replication. Intravenous inoculation of asexual NF 54 cell bank parasites increased the ability of NF54 to bind to CD 36 receptors. Scanning electron micrographs indicated that NF54 host passage had resulted in an increase of knobs in the membrane of the infected erythrocyte [57]. However, the relative importance of epigenetic reprogramming could not be assessed because asexual parasites were used for inoculation of the human volunteers. The importance of epigenetic reprogramming and host passage was investigated in parasites from the Tübingen controlled human malaria infection trial 1 [58]. NF54 passage through mosquito and human host resulted in a complete shift in *var* gene transcription compared to the premosquito [59] culture. Subsequent *in vitro* tissue culture growth showed that cultures from intradermally and intravenously infected individuals transcribed the same *var* genes but that the transcription signals were higher in parasites recovered after intradermal sporozoite inoculation [32]. Parasites from intradermal sporozoites infections showed higher surface signals after incubation with the MOA serum than parasites from intravenous infections. This indicates that the surface signal of the MOA serum is correlated with the transcription strength of individual *var* genes in the NF54 genetic background. Consistent with this, the decrease in the *var* gene transcription signal of the NF54 PfEMP1 knock-down clones delta C2 and delta E5 resulted in a decrease of the MOA serum surface signal. However, in the MOA PfEMP1 knock down clone, a decrease in the *var* transcription signal was not associated with a decrease in the surface signal.

Immuno-electron microscopy of parasites incubated with the MOA serum showed that in the MOA clone D2 and in the NF54 clone E5 gold particles bound to membrane areas with and without knobs. The surface signal of the MOA clone D2 and the NF54 clone E5 was removed by trypsinisation, confirming the surface localisation of these antigens in both genetic backgrounds. In contrast, *var* gene transcription knock down only affected the surface signal of the NF54 clone. Collectively the data suggest that the VSA surface signal of parasites from a chronic infection is predominately composed of trypsin-sensitive non-PfEMP1 VSAs, while in NF54 parasites the majority of the VSA surface signal is due to trypsin-sensitive PfEMP1 VSAs.

Of the *P*. *falciparum* VSAs, STEVOR, RIFIN, SURFIN and PfEMP1 have all been shown to be sensitive to trypsinisation, but the importance of the non-PfEMP1 VSAs for antigenic variation and as surface antigens has been as issue of scientific debate. Recently, it has been shown that the largest non-PFEMP1 VSA families STEVOR and RIFIN are displayed on the surface of the infected erythrocyte and that they mediate adhesion between infected erythrocytes, a phenomenon referred to as rosetting [60–62] by binding to glycophorin C and blood group A. Importantly, blood group A is also expressed on endothelial cells, suggesting a mechanism by which RIFINs could mediate cytoadhesion [62]. The importance of non-PfEMP1 VSA as antigens has recently been strongly supported by a study characterizing cross reactive antibodies from 2 semi-immune Kenyan individuals [63]. The authors identified highly reactive components of semi-immune sera through agglutination assays with multiple *P*. *falciparum* strains and subsequently characterized the antibodies with broadest reactivity by generating immortalized B Cells from the donors. Western blot analysis of 3D7 parasites enriched for binding to monoclonal cross reactive antibodies identified RIFIN proteins as the primary target of the antibodies.

Is there any seroepidemiologic evidence supporting the hypothesis that PfEMP1 may not be the only VSA expressed on the surface of infected red blood cells? Previous investigations in Gabon have indeed shown that the antibody repertoire against RIFINs expands with increasing age and that it represents a larger fraction of the total adult anti-VSA reactivity then anti-PfEMP1 antibodies [64,65]. A study from Papua New Guinea investigated the age dependent acquisition of antibodies against recombinant DBLs from naturally circulating parasite populations. Antibody reactivity against the recombinant DBL proteins peaked at age 3–4, but subsequently plateaued. Analysis of antibody reactivity against VSA in the same populations showed that it continued to increase with age throughout adulthood, suggesting that VSAs are not exclusively composed of PfEMP1 [66]. Previous investigations employing sera and parasites from children and adults have shown that strain transcending VSA recognition develops within the first years of life [5,6,37]. Seroepidemiological studies based on the recombinant DBL-tags of the 3D7 *var* gene repertoire demonstrated that children develop anti-PfEMP1 immunity in a sequential fashion against UpsA, UpsB and UpsC *var* gene products during childhood [39]. These results have been further supported by a study assessing surface reactivity from Kenyan individuals with complicated and uncomplicated malaria against wild type and *var* knock down parasites of the NF54 clone 3D7 [40]. Surface reactivity against 3D7 wild type parasites was consistently higher than in 3D7 *var* knock down parasites and increased with age. However, sera from semi-immune individuals still exhibited IgG antibody binding in the 3D7 *var* knocks down parasites, suggesting that non-PfEMP1 VSAs were also targeted by the antibodies. A recent analysis of transcription data from field isolates suggests that anti-PfEMP1 antibodies not only select against specific PfEMP1 variants, but also against the amount of PfEMP1 on the surface of iRBCs in chronic infections [67]. The trypsin sensitive non-PfEMP1 VSA surface reactivity observed in the MOA clones is clearly consistent with these observations.

Our results raise a few fundamental questions with respect to *var* gene expression in different *P*. *falciparum* isolates. Differences in the strength of transcription signals between laboratory strains and field isolates suggest that wild type *var* promoters from field isolates may have a strong epigenetic imprint that contributes to the strong transcription activity and to the observed low switching propensity. We have shown before that in transgenic parasites the transcription activity of a subtelomeric recombinant *var* gene promoter can be increased markedly by the strength of applied drug selection [30]. At the same time this is associated with a lower switching propensity, suggesting that epigenetic modifications can influence the transcription activity and switching propensity of a recombinant *var* promoter. In a recent investigation of NF54 parasites recovered from controlled human infections [58], we have shown that longer in-host replication results in parasite populations with strong *var* gene transcription signals and low switching propensities [32].

In the MOA patient, semi-immunity likely exerted strong selective pressures on parasite replication and consequently parasites transcribing *var* genes with low off rates for these loci might have been at a selective advantage. The low switching propensity observed in the *in vitro* MOA parasites is clearly consistent with strong selective pressure favouring parasite populations transcribing specific *var* genes.

Recombination has been shown to occur amongst different members of the *var* gene families and it has been proposed that this is a mechanism to generate chronic infections [48]. We tried to address this question by designing gene specific primers for MOA DBL *var* sequences [30,68] a method that we have previously employed to confirm recombination events with the DBL alpha region [47]. We found no evidence for the generation of new chimeric sequences at different time points of the *in vivo* infection. Because our analysis is limited to the DBL alpha region, it does of course not exclude recombination in other regions of the *var* genes. We noted the absence of one *var* DBL in a subpopulation of MOA clones, which is consistent with previous reports of larger recombination during *in vitro* growth [48,49]. However, it is important to note that so far molecular epidemiologic investigations of *var* gene diversity have been performed mostly on the DBL alpha region [69,70]. The relative importance of recombination events for chronic infections therefore is currently not clear.

How could the presence of PfEMP1 in the membrane of the infected RBCs in a long term infection be reduced, despite a very strong *var* gene transcription signal? Here it is important to realize that although the MOA serum clearly exhibited broad anti-PfEMP1 reactivity, it is still possible that individual PfEMP1 variants were not detected by it. Indeed it has been described that IgM masking can prevent the development of PfEMP1 variant specific IgG antibody responses [71]. To definitively assess the relative contribution of the different VSAs to the surface signal of the MOA clones, investigations with VSA family specific monoclonal antibodies are necessary. The transmission electron microscopic experiments performed in this work detected no obvious difference with respect to knobs in the membrane of RBCs infected with NF54 or MOA clones, suggesting that knob architecture *per se* was not different in these strains. However, alterations in proteins that mediate PfEMP1 transport such as MAHRP1 could equally result in the observed phenotypes [72].

The data in this manuscript suggest that the VSAs responsible for the surface signal of parasites from a chronic infection differ from VSAs responsible for the surface signals in CD36 selected laboratory parasites. This is consistent with a previously proposed model that differentiates between VSAs expressed on parasites from individuals with severe malaria (VSA_SM_) and VSA expressed on parasites from individuals with uncomplicated malaria (VSA_UM_) [73,74]. According to this model, expression of VSA_SM_ may confer a growth advantage in children, generating high parasitemias and possibly a strong humoral immune response. During infections of semi-immune individuals this restricts the growth of parasites expressing VSA_SM_, resulting in growth of parasites expressing VSA_UM_ that in turn replicate in a less efficient manner [75,76]. There is strong evidence that UpsA *var* genes may be part of VSA_SM_ because they mediate disease associated phenotypes such as binding to endothelial protein C receptor [77–81]. Studies assessing *var* gene transcription in children with uncomplicated malaria have consistently shown preferred transcription of Ups C and Ups B genes [82,83]. Given the importance of trypsin-sensitive non-PfEMP1 VSAs in the MOA parasites, we hypothesize that RIFIN and STEVOR proteins may be part of the VSA_UM_ expressed on the surface of infected red blood cells during chronic infections.

Understanding semi-immunity to blood stage parasites is critical for the development of a malaria vaccine. The work presented in this manuscript suggests that phenotypic and genetic analyses in culture adapted field isolates with serum from the individual patients can provide new insights that cannot be gathered from work with laboratory strains alone. Furthermore, controlled human infections of semi-immune individuals with NF54 parasites can provide new insights into the role of semi-immunity for VSA expression. Over the last years, PfEMP1 has emerged as a candidate for a vaccine targeting the blood stages of *P*.*falciparum*. The role of non-PfEMP1 VSAs for semi-immunity will have to be determined in future investigations.

## Material and Methods

### Ethics statement

The antigenic diversity study was approved by the local ethics committee in Lambaréné, Gabon: CERMEL (Centre de Recherche Médicale de Lambaréné), Lambaréné, Gabon. Written informed consent has been provided by all volunteers. The study followed the principal of the Declaration of Helsinki in the 6^th^ revision as well as Good Clinical Practice (ICH-GCP).

### Study protocol of the antigenic diversity study

All samples of the MOA individual investigated in this study were obtained as part of the antigenic diversity study in Lambaréné in Gabon from June 2006 until May 2007 [30,84]. This study included semi-immune asymptomatically *P*. *falciparum* infected adults in an area with holoendemic malaria transmission. The study was approved by the local ethics committee. Inclusion criteria were: age of 18 years or older, microscopic evidence of *P*. *falciparum* parasitemia and absence of symptoms. Exclusion criteria were: age less than 18 years, pregnancy, symptoms or signs of malaria. The participants were followed for a total of 70 days and stayed untreated throughout the study period as long as they were asymptomatic. Blood and filter paper blood spots for PCR analysis were obtained twice per week for parasitemia assessment. Blood was drawn once per week for storage of serum, RNA in Trizol, RBC pellets and glycerol cryopreservation of malaria parasites. Parasitemia was assessed every three to four days by thick blood smear according to the Lambaréné method [85]. Extraction from filter papers was performed at the medical research unit of the Albert Schweitzer Hospital. DNA and all other specimens were transported to the Institute of Tropical Medicine at the University of Tuebingen, Germany. A total of 1017 individuals were screened and 36 individuals with microscopic parasitemias were identified. Based on *mspII* allele typing two clonal infections were identified. The asymptomatic clonal chronic infection of the 18 year-old male individual "MOA", was investigated further in this work. He became symptomatic with a new *P*. *ovale* infection on day 42 and was treated according to local guidelines. Blood draws were continued until day 70 of the investigation.

### Parasite lines and culture

The field isolate MOA bulk represents cryopreserved *P*. *falciparum* parasites obtained from the MOA individual at day 7 of the 70 day sampling period. *In vitro* culture adaptation was performed in Tuebingen. Parasites were cultivated as reported elsewhere [30]. The parasites were kept in tissue culture, using 0+ blood from the local blood bank, for 25 generations prior to cloning by limiting dilution. Two limiting dilution experiments of MOA bulk were performed. The first one generated the 3 clones C3,D2 and D5 [30]. For the current manuscript a second limiting dilution experiment of the original MOA bulk culture generated 16 new clones (G3,E8,G9,B5,C4,E10,H4,G2, D11,F11,A1,H6,E1,B10,C8,J1). This resulted in a total of 19 MOA clones. At the time point of clonal *var* transcription analysis, the 16 MOA clones generated in the second limiting dilution experiment had been in clonal culture for approximately 35 generations (time since limiting dilution). The clones D2, D5 and C3 had been in continuous tissue culture for > 65 generations (time since limiting dilution). E5 is a sibling parasite of 3D7 that shares 36 *var* genes with 3D7, but also carries 13 different *var* genes [47]. NF54-C2 is isogenic with 3D7 and was isolated from a bulk NF54 culture by limiting dilution [31].

### DNA extraction and genotyping

DNA was extracted from filter paper blood spots, red blood cell pellets of the MOA individual and *in vitro* cultured parasites with the QIAamp DNA blood Midi Kit (Qiagen) according to the protocols provided by the manufacturer. *msp1* and *msp2* allele genotyping was conducted by standard nested PCR employing primers and conditions published elsewhere [86].

### Characterization of *var* gene DBL sequences from DNA and cDNA

The *var* gene repertoires of the individual MOA D2, D5 and C3 clones were determined with a PCR cloning approach using the universal *var* primers αAF (5’-GCACG(A/C)AGTTTTGC-3’) and αBR (5’-GCCCATTC(G/C)TCGAACCA-3’) on genomic DNA [46]. PCR products were cloned in *E*. *coli* (TOPO TA cloning kit pCR 2.1-TOPO Vector, Invitrogen) according to standard procedures and DNA was extracted using the Qiagen Miniprep kit. Plasmid sequences were analysed and aligned with BioEdit software (Carlsbad, California). The same method was used to analyse the *var* repertoire in genomic DNA from blood samples of the MOA individual. We used blood samples of day 0, day 7 and day 28 of the study. Cloning of *var* sequences obtained by universal *var* primers was also performed on copy DNA to identify the active *var* gene in RNA from tissue culture adapted field isolates (D2, D5 and C3 [30]) and *in vivo* RNA obtained from blood samples. In 9 of the 16 clones isolated in the second limiting dilution experiment (C8, G9, E1, B10, D11, F11, H6, A1, G2) the dominant *var* transcript was characterized by cDNA PCR with the universal *var* primers αAF and αBR followed by PCR fragment sequencing (see below). Fragment specific primer pairs were designed for all sequences (S2 Table). In this publication, differing from Enderes et al. [30], we named DBL MOA D5-D5 as PD5, MOA D2-D2 DBL as PD2, MOA C3-C3 as PC3. All primer pairs were validated by serial dilution of genomic DNA, PCR fragments were analyzed by Sanger sequencing prior to use by quantitative Real-Time PCR (see below).

### RNA extraction and cDNA synthesis

A Sorbitol synchronisation of 20 ml parasite cultures, RNA extraction and cDNA synthesis was done as described in [30]. Possible DNA contamination in the cDNA was tested by the evaluation of proper splicing of the gene PFD1155w as described before [31].

### Analysis of *var* gene *in vivo* transcription analysis by DBL cloning

To analyse *var* transcription *in vivo*, Trizol-conserved RNA from the MOA individual was analysed. cDNA was synthesized by two different approaches: one employing random primers and the other using the universal *var* primers αAF and αBR. The cDNA was analysed by PCR cloning as described above. Transcript quantification was done by counting the number of times that the individual sequences were obtained in the two cloning experiments. In both cloning experiments d0_37 was the most frequent transcript.

### *In vitro* analysis of *var* gene transcription by quantitative Real-Time PCR and cDNA PCR fragment sequencing

DBL specific MOA primer pairs for all the 36 DBLs of the culture adapted MOA parasites were designed and subsequently validated on serial dilutions of MOA clone genomic DNA by Real-Time PCR followed by DNA sequencing of the PCR product as described previously [30]. The 16 MOA clones generated in the second limiting dilution experiment underwent a first transcription analysis with 33 primer pairs obtained from the DBL cloning experiments with the clones D2,D5 and C3 as well as the 6 *in vivo* transcripts. This primer set detected a dominant transcript in 7 (H4, G3, E8, B5, J1, E10 and C4) of the 16 clones. However in 9 clones (C8,G9,E1,B10,D11,F11,H6,A1,G2) no dominant transcript was detected. In these 9 clones cDNA PCR with the universal primer αAF and αBR followed by PCR fragment sequencing was employed to characterize the dominant transcript. This detected the *in vivo* DNA sequences (D0_36, D7_15 and D7_33) as the dominant transcripts in 9 clones. We subsequently designed DBL specific primers (T0_36, D7_15 and D7_33) and quantified the transcription signal by qRT PCR. To assess the possible presence of *var* transcripts not covered by the 36 gene specific primers we also performed PCR with the degenerate DBL primers on the clones in which a dominant transcript was detected with the initial 33 primer set. Specific *var* gene primer correction factors to correct for primer efficiency relative to the amplification of the housekeeping gene arginyl-tRNA synthetase (PFL0900 c) were assigned for all primers and ΔCt was calculated accordingly (User bulletin 2, Applied Biosystems). *var* gene transcription of NF54 was assessed as described previously [21,22,30]. Determination of the E5-specific *var* sequences was described previously [47]. Gene-specific primers were designed for 13 sequences (S2 Table) and validated on serial dilution of E5 genomic DNA. Transcription of *bsd* (blasticidin S deaminase) in PfEMP1 knock-down strains was assessed with the primers *bsd* fwd 5’-TTGTCTCAAGAAGAATCCAC-3’ and *bsd* rev 5’-TCCCCCAGTAAAATGATATAC-3’ [22,30].

### Generation of transgenic *var* knock-down parasites

Plasmid pV_C_BB/IDH [22] was utilized to generate transgenic *va*r knock-down parasites in the MOA clone (MOA D2) as well as in NF54-C2 and E5. Here, expression of the transgene *bsd* is driven by an UpsC promoter that is paired with an intron promoter driving human *dhfr* (dihydrofolate reductase) gene. The knob-positive NF54-C2 clone was transfected with the plasmid pV_C_BB/IDH. Transfection was conducted as described in Deitsch et al. [87]. Plasmids were propagated in *E*. *coli* and isolated with the Plasmid Maxi kit (Qiagen). Uninfected erythrocytes were loaded with plasmid DNA by electroporation and double sorbitol synchronised parasites were added. Subsequently, parasites were cultured for 4 generations in 2.5% hematocrit of loaded erythrocytes. Due to the high multiplication rate, the cultures were diluted every growth cycle with newly plasmid loaded erythrocytes. On day 9 of the experiment, the drug WR 99210 (5nM) was added to ring stage parasites of the transfectants in the MOA genetic backgrounds. Transfectants in the E5 genetic background were immediately selected with blasticidin because E5 already carries a *hdhfr* expression cassette [21]. Shut down of MOA D2 *var* expression in stably transfected parasites was induced by application of 20μg/ml blasticidin (final concentration). Episomally transfected ΔMOA D2 carrying the pV_C_BB/IDH plasmid was selected with increasing blasticidin pressure starting at 1μg/ml up to 20μg/ml. Transcription of blasticidin S deaminase and absence of transcription of endogenous *var* genes was quantified by Real-Time PCR on cDNA as described above.

### CD36 receptor binding selection

Human melanoma C32 (ATCC^®^, CRL-1585^™^) cells were cultured in DMEM (PAA Laboratories) supplemented with 10% FBS (PAA), 1% non essential amino acid solution (Sigma-Aldrich), 0,05 mg/ml Gentamicin (PAA), and 2 mM L-Glutamine (PAA). To quantify binding of parasitized red blood cells to the C32 cells microscopically, a cover slip was inserted into the C32 culture flasks. Trophozoite enrichment was performed with MACS columns (Miltenyi Biotec). Comparable (~10x10^7^) numbers of parasites were diluted in binding medium (450ml H_2_0, 2.98g HEPES, 5.2g RPMI without glutamine and NaHCO3, 10% ml human serum 0^+^), transferred into the C32 cell culture bottle (25cm^2^) with ~80% confluency and incubated at 5% CO_2_ for 2h at 37°C. Unbound trophozoites were removed by washing with binding medium 3 times. The cover slip was removed from the culture flask with a forceps, fixed in 2% glutaraldehyde, stained with 5% Giemsa stain (Merck). The number of bound trophozoites per 50 C32 nuclei was determined by light microscopy. Subsequently, parasite medium and uninfected red blood cells (uRBCs) were added and the culture was incubated overnight in the parasite incubator. The following day the parasites were recovered in ring stage.

### Flow cytometry analysis

Parasites were synchronised with MACS (Miltenyi Biotec, order no: 130-042-901). The schizont pellet was mixed with uRBCs to obtain a parasitemia between 5–15%. 12μl of parasite pellet were stained with Hoechst 33342 (0.01mg/ml) for 30min at 37°C under tissue culture conditions. After washing three times with 1x phosphate buffered saline (PBS) the pellet was incubated with MOA serum (1:10 in PBS) of day 70 of the study. The infected red blood cells (iRBCs) were washed three times and the pellet was stained with goat anti-human IgG (Southern Biotech) (1:200 in PBS) for 30min. After washing,the pellet was incubated with rabbit anti-goat fluorescein isothiocyanate (FITC) conjugated IgG (1:50 in PBS) (Southern Biotech) for 30 min. After washing and dilution with 1 ml PBS the cells were measured in a FACS CantoII (BD). FACS (Fluorescence-activated cell sorting) was performed as described elsewhere [88,89]. All experiments were conducted at least in triplicates with uRBCs as control. Mean fluorescence intensity (MFI) for each tube was defined as: MFI = MFI_iRBCs_−MFI_uRBCs_. The CD 36 selected ΔE5E2 parasites and the blasticidin selected ΔE5E2 were used as positive and negative controls in all FACS experiments with the MOA clones.

### Trypsinisation of erythrocyte surface proteins

Trypsinisation of *P*. *falciparum* trophozoites following MACS separation was done as described elsewhere [90].

### Electron microscopy

Immune labeling was done using trophozoites purified from a 10ml- *P*. *falciparum* culture with MACS^®^ (Miltenyi Biotec) and washed with 1x PBS. The cells were pelleted in a low-binding Eppendorf tube (Biozym) and and incubated with MOA serum of day 70 for 30 minutes, followed by 2–3 washing steps using SafeSeal Vertex tips (Biozym) with 1x PBS. Goat anti human IgG (Southern Biotech) was diluted 1:200 in 1x PBS prior to incubation with the pellet for 30 minutes. Then the mixture was washed 2–3 times with 1xPBS and the cells were stained with Gold-AffiniPure Donkey Anti-Goat IgG 12 nm Gold (dianova, 1:20) for 30 minutes before 2–3 washing steps. The cell pellet was chemically fixed (4% formaldehyde, 2.5% glutaraldehyde in 0.1M phosphate buffer pH 7.4), cryo-protected with 30% glycerol in water and cryo-fixed by high-pressure-freezing in a Balzers HPM-010. For subsequent freeze substitution, embedding, sectioning and contrasting and we followed the protocol described in Moussian et al. [91]. The sections were examined with a Tecnai G2 Spirit (FEI, Eindhoven, Netherlands) operated at 120kV transmission electron microscope that was equipped with a Gatan Ultrascan 4000 CCD-camera (Gatan, Pleasanton, CA, USA). Micrographs were recorded with the manufacturer’s software at 4k x 4k resolution. For analysis by scanning electron microscopy cells were fixed with 0.5% glutaraldehyde, 2% paraformaldehyde mounted on polylysin-coated coverslips followed by another fixation step with 2.5% glutaraldehyde. The erythrocytes were postfixed with 1% osmium tetroxid in water, dehydrated in a graduated series of ethanol followed by critical-point-drying with CO2 in a Polaron critical-point-dryer. Finally the cells were sputter coated with a 6nm layer of platinum (Bal-Tec MED 010) and examined with a Hitachi S-800 field emission scanning electron microscope at an accelerating voltage of 15 KV. Knob quantification of the D2 and D5 clones was performed by two independent investigators (EB and MF) in a blinded fashion. TEM cuts of 8–10 iRBCs per clone were examined and the number of knobs counted.

### Statistical analysis

The heat map was generated in R [92] using the package *lattice* and *latticeExtra*, and post-edited in GIMP 2.8.14. The individual MOA clones and the MOA bulk were ordered by size according to the strength of their flow cytometry signal (FACS). *var* gene expression signals for each of the 36 loci were expressed as percentage of total *var* signal of the individual clone. The strength of the expression signal of the individual *var* gene was colored in accordance with their magnitude using user-defined steps for color graduation in the R heat map. Two data points are missing (D7_15 and D7_33 for MOA F11). T-tests to calculate the statistical significance of data were analyzed with Microsoft Excel.

## Supporting Information

S1 Fig *in vitro var* gene transcription analysis of MOA parasites. Long term transcription profiling with gene specific primers for day 0 *var* transcripts in culture adapted MOA bulk parasites for a total of 150 generations of continued growth. Transcript d0_37 was the most abundant transcript *in vitro*. (A), (B) and (C) display *var* transcription after 30, 90 and 150 generations of *in vitro* growth.
(TIF)

S2 Fig MOA Clones (35 generations after cloning) transcribing T0_36 display surface signals from high to low. (A) Clone G2 exhibits the strongest transcription signal and has a medium MFI of 69.67. (B), (C) and (D): The clones C8, A1 and H6 transcribe T0_36 at identical strength, yet the surface signal for clone C8 and A1 is high (MFI of 243 and 258.33) and low for clone H6 (MFI of 48.67, lowest surface signal in the entire population). (E) Clone F 11 transcribes T0_36 at high levels yet has medium surface signal (MFI of 81.67).
(TIF)

S3 Fig MOA clones (35 generations after cloning) show low transcription signals but high to low FACS signals. (A) The clone E1 displays the lowest transcription signal yet has a high surface recognition signal (MFI of 161.67). (B) and (D) The clones D11 and G9 both transcribe DBL D7_35 at low levels yet have medium and low surface reactivity respectively (MFI of 62 and 53.75).
(TIF)

S4 Fig MOA serum surface recognition of the laboratory strain NF54-C2 is increased after CD36 receptor binding. (A) The copy number is shown at the y-axis. The adhesion phenotype (bound trophozoites per 50 C32 cell nuclei) is depicted on the right and also the flow cytometry dot plot, where iRBCs (right lower corner) are not recognized by the antibodies of MOA day 70 serum. (B) Panning for CD36 binding resulted in a strong adhesion phenotype and an upward shift of the infected erythrocyte population in flow cytometry with MOA day 70 serum. Binding of the trophozoites (dark dots) to a C32 cell is demonstrated in the light microscopy picture below.
(TIF)

S5 Fig PfEMP1 knock-down in NF54 clone C2 is efficient and can be reversed by CD36 binding. (A) Removal of blasticidin and selection for CD36 binding evokes *var* gene activation and cytoadhesion and yields a positive signal in flow cytometry. (B) Knock-down of PfEMP1 efficiency shown by transcription profiling. There is no adhesion to the CD36 receptor (right graph) and iRBCs are not recognized by MOA day70 serum (dot plot). (C) Western Blot demonstrating efficient PfEMP1 knock-down. Uninfected erythrocytes served as control. Cell lysates were generated using Triton X-100, run on a tris acetate gel, blotted on a nitrocellulose membrane and stained with the PfEMP1-specific antibody α-ATS. α-ATS detects PfEMP1 (marked with an asterisk) in CD36-selected ΔE5E2, but not in its PfEMP1-knock down cell line ΔE5E2+20μg/ml bsd. The transfected ΔNF54+20μg/ml clone also does not have a band for PfEMP1. Cross-reaction with the cytoskeletal protein spectrin is seen at ~250kDa in all samples. The columns were rearranged for clarity (dotted line).
(TIF)

S1 Table DBL specific primers for amplification of DBLs from DNA of days 0, 7 and 28. Sequence D0H21 was identified by DBL cloning on all three days and was used as a control for primer design. All chimeric DBL sequences and the corresponding primers can be supplied by the authors’ upon request.
(DOCX)

S2 Table *var* gene specific PCR primer set for MOA culture adapted field isolates and *in vivo var* transcripts. Forward and reverse sequences for the 36 *var* loci were designed based on the corresponding DBLα sequences. MOA clone names are indicated by capital letters. Day0 *in vivo* MOA transcripts are indicated by the prefix d0. DBL T0_36 is the same as DBL D7_87 (KC887630) and C3_42 (KC887685) in the NCBI database.
(DOCX)

S3 Table Transcription strength of the dominant DBLs in all 19 MOA clones and the MOA bulk culture. (DOCX)

S4 Table Gene specific PCR primer pairs for E5 *var* sequences. Primer pairs were designed based on the hypervariable regions of the E5 specific DBLs.
(DOCX)
