# Supplementary figures and images for: In Vitro Variant Surface Antigen Expression in Plasmodium falciparum Parasites from a Semi-Immune Individual Is Not Correlated with Var Gene Transcription
Source: PLoS One. 2016 Dec 1;11(12):e0166135. doi: 10.1371/journal.pone.0166135 (PMC5132323; doi:10.1371/journal.pone.0166135)

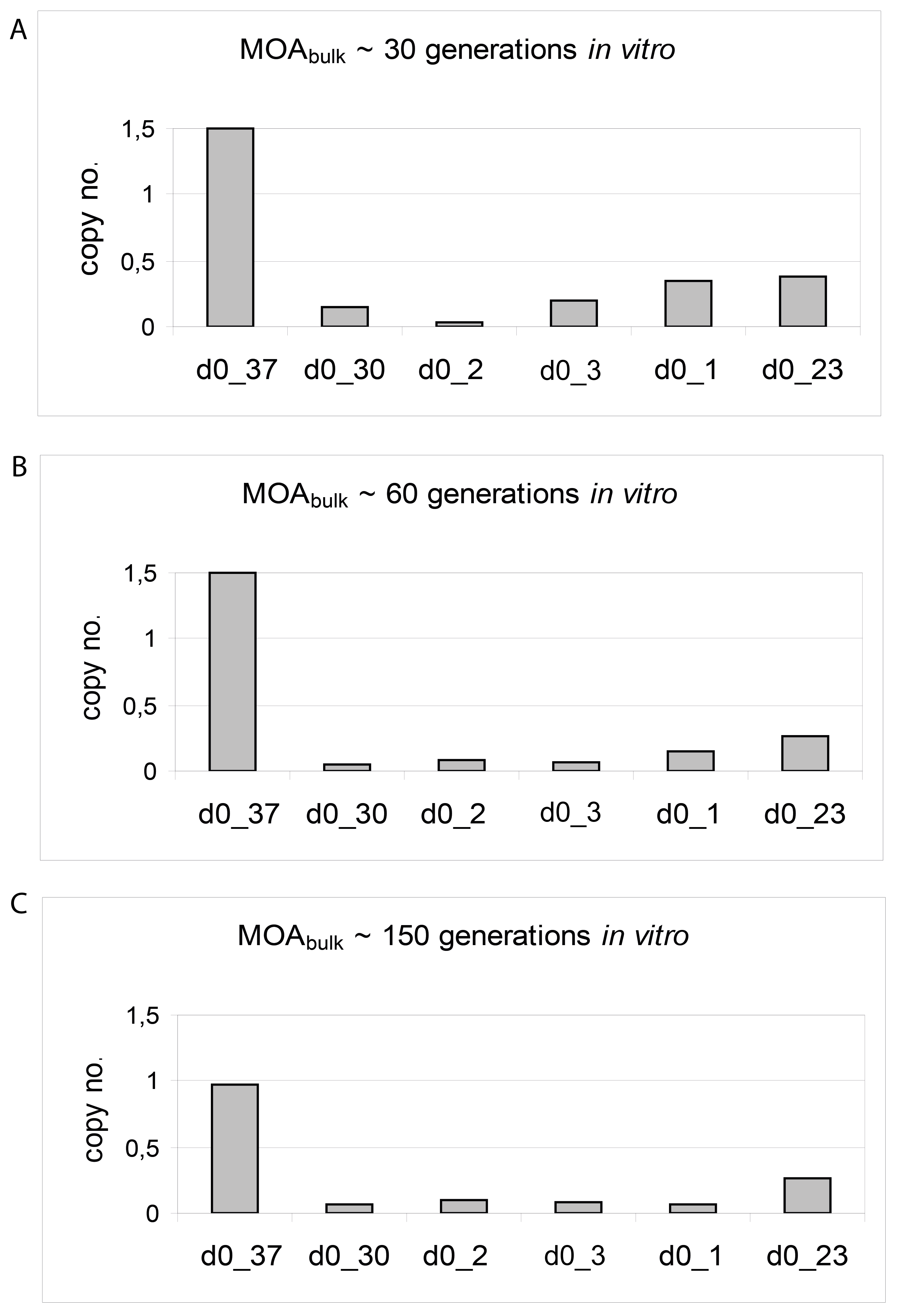

Supplement: S1 Fig — Long term transcription profiling with gene specific primers for day 0 var transcripts in culture adapted MOA bulk parasites for a total of 150 generations of continued growth. Transcript d0_37 was the most abundant transcript in vitro. (A), (B) and (C) display var transcription after 30, 90 and 150 generations of in vitro growth. (TIF) [file pone.0166135.s001.tif]

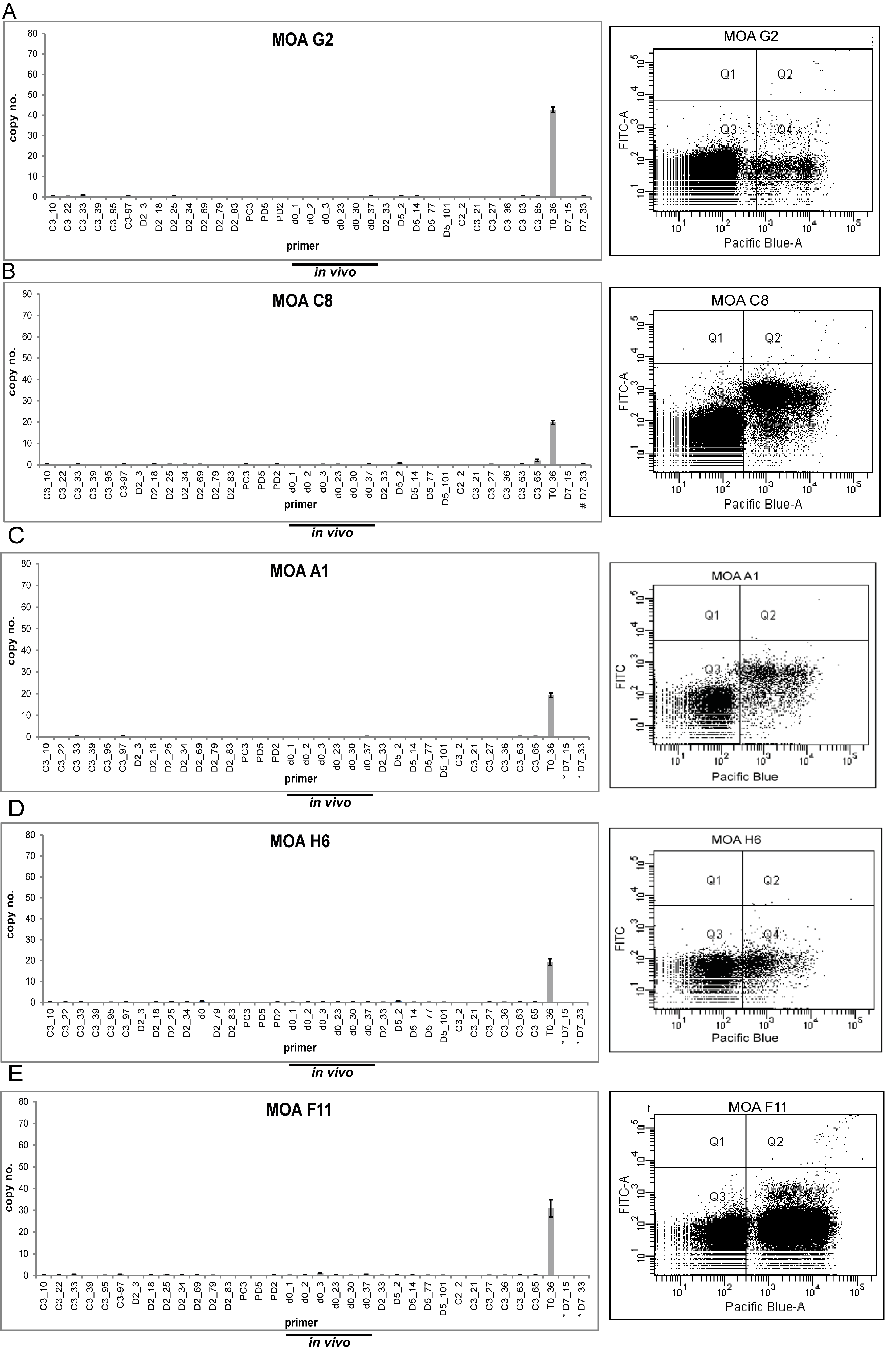

Supplement: S2 Fig — (A) Clone G2 exhibits the strongest transcription signal and has a medium MFI of 69.67. (B), (C) and (D): The clones C8, A1 and H6 transcribe T0_36 at identical strength, yet the surface signal for clone C8 and A1 is high (MFI of 243 and 258.33) and low for clone H6 (MFI of 48.67, lowest surface signal in the entire population). (E) Clone F 11 transcribes T0_36 at high levels yet has medium surface signal (MFI of 81.67). (TIF) [file pone.0166135.s002.tif]

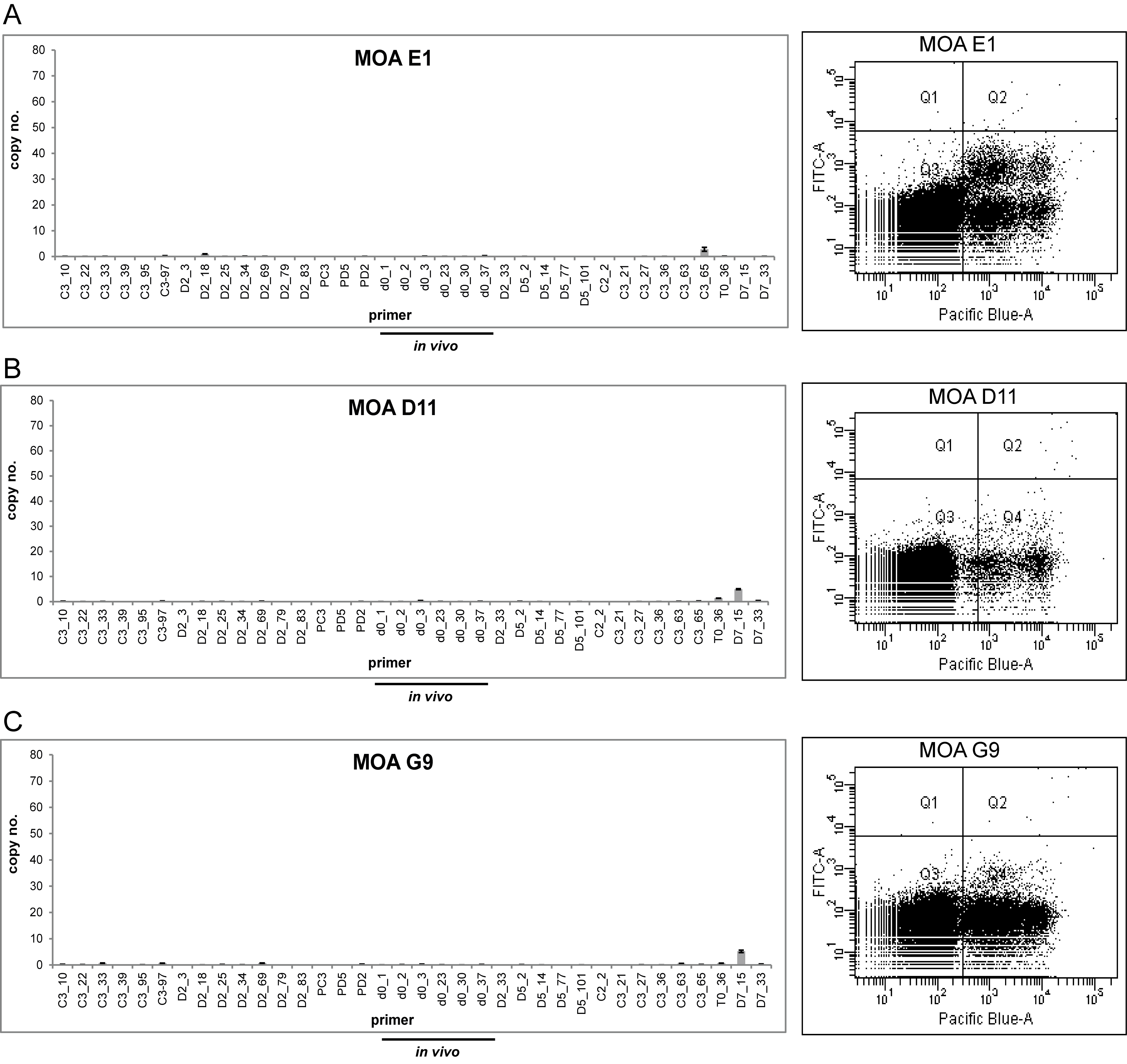

Supplement: S3 Fig — (A) The clone E1 displays the lowest transcription signal yet has a high surface recognition signal (MFI of 161.67). (B) and (D) The clones D11 and G9 both transcribe DBL D7_35 at low levels yet have medium and low surface reactivity respectively (MFI of 62 and 53.75). (TIF) [file pone.0166135.s003.tif]

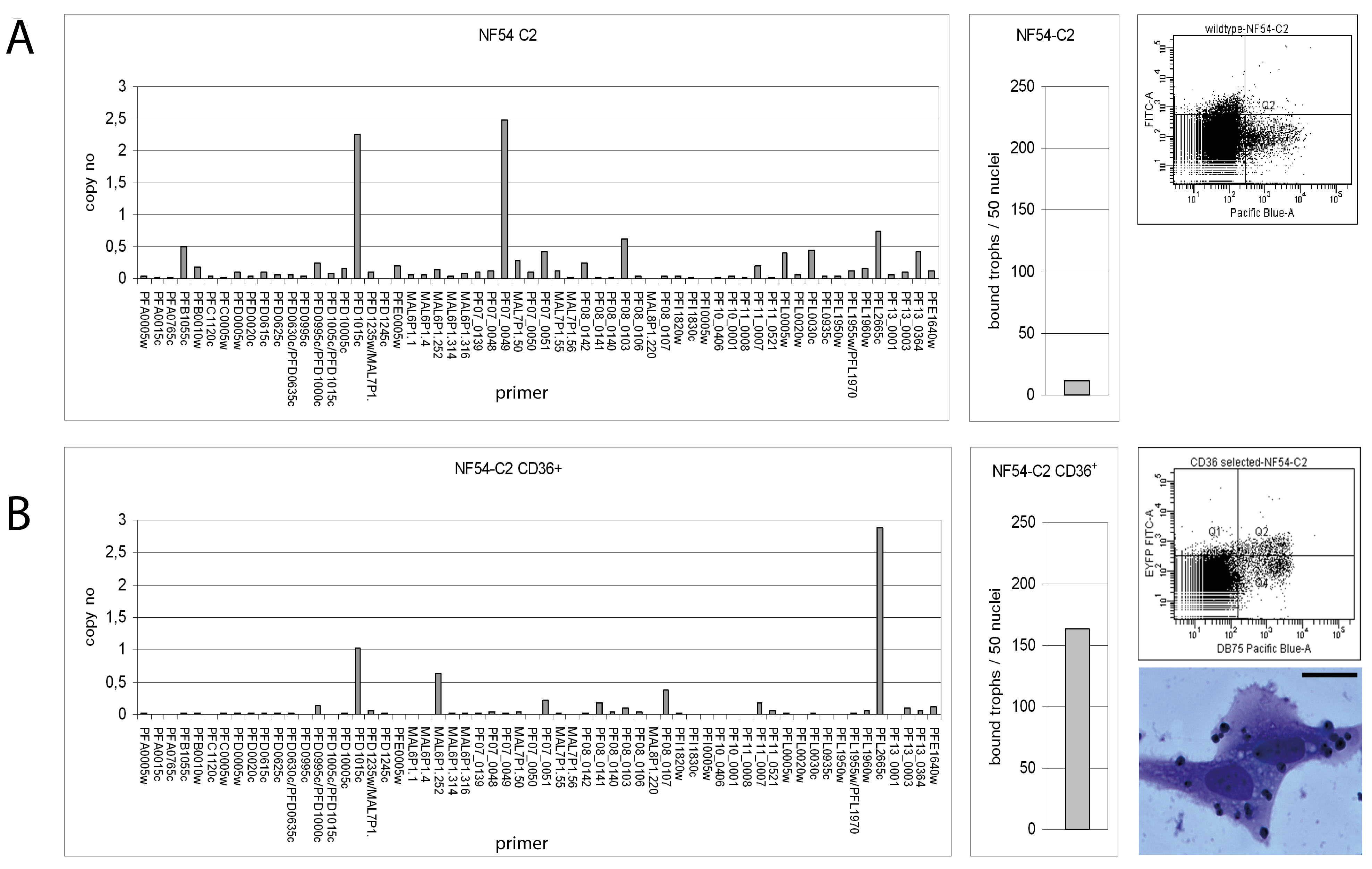

Supplement: S4 Fig — (A) The copy number is shown at the y-axis. The adhesion phenotype (bound trophozoites per 50 C32 cell nuclei) is depicted on the right and also the flow cytometry dot plot, where iRBCs (right lower corner) are not recognized by the antibodies of MOA day 70 serum. (B) Panning for CD36 binding resulted in a strong adhesion phenotype and an upward shift of the infected erythrocyte population in flow cytometry with MOA day 70 serum. Binding of the trophozoites (dark dots) to a C32 cell is demonstrated in the light microscopy picture below. (TIF) [file pone.0166135.s004.tif]

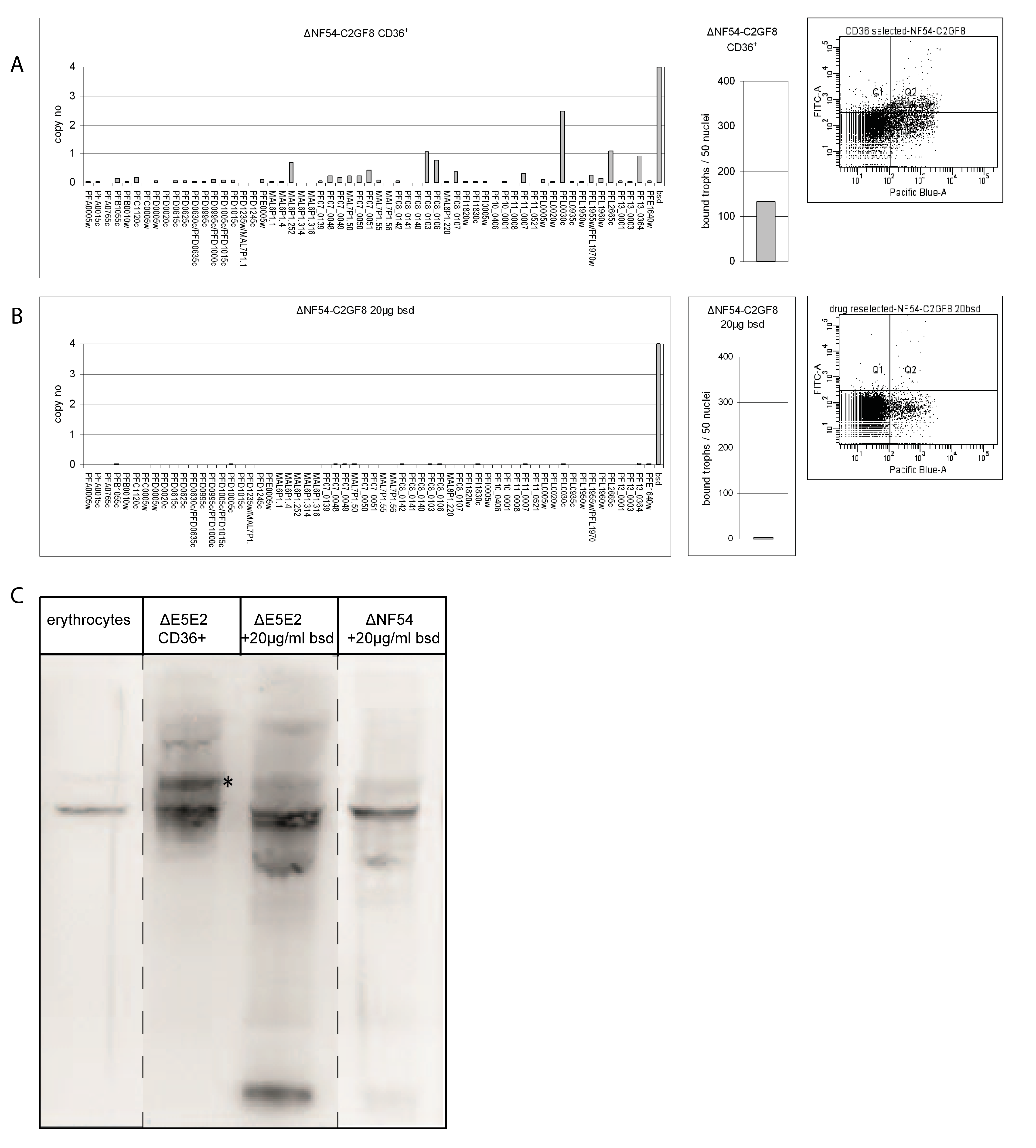

Supplement: S5 Fig — (A) Removal of blasticidin and selection for CD36 binding evokes var gene activation and cytoadhesion and yields a positive signal in flow cytometry. (B) Knock-down of PfEMP1 efficiency shown by transcription profiling. There is no adhesion to the CD36 receptor (right graph) and iRBCs are not recognized by MOA day70 serum (dot plot). (C) Western Blot demonstrating efficient PfEMP1 knock-down. Uninfected erythrocytes served as control. Cell lysates were generated using Triton X-100, run on a tris acetate gel, blotted on a nitrocellulose membrane and stained with the PfEMP1-specific antibody α-ATS. α-ATS detects PfEMP1 (marked with an asterisk) in CD36-selected ΔE5E2, but not in its PfEMP1-knock down cell line ΔE5E2+20μg/ml bsd. The transfected ΔNF54+20μg/ml clone also does not have a band for PfEMP1. Cross-reaction with the cytoskeletal protein spectrin is seen at ~250kDa in all samples. The columns were rearranged for clarity (dotted line). (TIF) [file pone.0166135.s005.tif]
