# Supplementary material for: In Vitro Variant Surface Antigen Expression in Plasmodium falciparum Parasites from a Semi-Immune Individual Is Not Correlated with Var Gene Transcription
Source: PLoS One. 2016 Dec 1;11(12):e0166135. doi: 10.1371/journal.pone.0166135 (PMC5132323; doi:10.1371/journal.pone.0166135)
Supplement: S1 Table — Sequence D0H21 was identified by DBL cloning on all three days and was used as a control for primer design. All chimeric DBL sequences and the corresponding primers can be supplied by the authors’ upon request. (DOCX) [file pone.0166135.s006.docx]

S1 Table: **Gene specific PCR primers for confirmation of *in* *vivo* *var* sequences obtained through DBL shotgun cloning**

| **DBL name** | **Forward primer** | **Reverse primer** |
| --- | --- | --- |
| **D28H13** | 5'-AATATATATGACAATTTGGTGG-3' | 5'-GGCTTGTCGTCACATCGGCATTT-3' |
| **D28L2F** | 5'-TTAGAAACAATGTTTGAGAACAT-3' | 5'-CTCGCGCATCGCATGTGATAGCTT-3' |
| **D28_23** | 5'-TTCGGGATAATATATGAAAAATTGGATG-3' | 5' CCCATGCCCTTTTGAACATGTTT 3' |
| **D28_18/d7_2** | 5'-AATACATAAGGACGTGACGAGGAC-3' | 5'-CGCCGTTCCTGTACAACATGTG-3' |
| **D28_21** | 5'-TTTTCGAGAAATTATATGAAG-3' | 5'-CCCATATTAGAGTCTTTTGTA-3' |
| **D7_39** | 5'-ATAATGGATTGGATGCGAAGA-3' | 5'-CCATCAGTCGGACTTCTTCCTA-3' |
| **D7_CH13** | 5'-GGCAATTTGAACGGCGCAAAAG-3' | 5'-CGGCATTTGTCATTAGCTTGAGAA-3' |
| **D7_87** | 5'-GATGATTTGACGAAGGACAATGATAAT-3' | 5'-ACTGATACACGTACAATTCTTATGCGC-3' |
| **D7_CH15** | 5'-CGCCAAAATATATGGTACATTACCCGAA 3' | 5'-TTTGTTAGAACATCTCCACTATGGCAT-3' |
| **D0_42** | 5'-TTTTCAAGAAAATACATGGAAATTT-3' | 5'-ACACATATACATTTGTTAGTACTC-3' |
| **D0H21** | 5'-GAAGGGAGGGAAGAATGCGCA-3' | 5'-CACCATGATTGGTACAGTGGC-3' |
| **D0H22** | 5'-TGTTTAAGAACATTCAGAAAAATA-3' | 5'-ATGGCCGCAACGACCATGCGAAAAA-3' |
