# Supplementary material for: In Vitro Variant Surface Antigen Expression in Plasmodium falciparum Parasites from a Semi-Immune Individual Is Not Correlated with Var Gene Transcription
Source: PLoS One. 2016 Dec 1;11(12):e0166135. doi: 10.1371/journal.pone.0166135 (PMC5132323; doi:10.1371/journal.pone.0166135)
Supplement: S2 Table — Forward and reverse sequences for the 36 var loci were designed based on the corresponding DBLα sequences. MOA clone names are indicated by capital letters. Day0 in vivo MOA transcripts are indicated by the prefix d0. DBL T0_36 is the same as DBL D7_87 (KC887630) and C3_42 (KC887685) in the NCBI database. (DOCX) [file pone.0166135.s007.docx]

S2 Table: **MOA primer set**

| **Primer** | **fwd** | **rev** |
| --- | --- | --- |
| MOA C3_10 | 5‘-GCAACGCTCAGGGTTTTAAC-3‘ | 5‘-ACTGCCGATGTATCAATTTTAGTG-3‘ |
| MOA C3_22 | 5‘-TAAGGACGGTCCAGATTATTATC-3‘ | 5‘-CGACAACAAGCTAAGAGATGC-3‘ |
| MOA C3_33 | 5‘-TGGTGAACTGGAACGTCTTCC-3‘ | 5‘-CATGCAGCGCGGCCACGGGT-3‘ |
| MOA C3_39 | 5‘-GACCTTAAGGACCTTCATATTG-3‘ | 5‘-GCAATGCTCCAACAGATGCTTAT-3‘ |
| MOA C3_95 | 5‘-TGACAATTTGGTGGTTGCAGCA-3‘ | 5‘-GTGATGCACCAGAAAGTGCTCA-3‘ |
| MOA C3_97 | 5‘-CGAGTGGGAAGAAAGGGCAC-3‘ | 5‘-ACGGTTAACCGCGCCACAATC-3‘ |
| MOA D2_3 | 5‘-CGACGAAGGGCGCACAAAATTAC-3‘ | 5‘-CATGCAAGGCGGAGCAAAATG-3‘ |
| MOA D2_18 | 5‘-GTATCTCGGTAATGACGATGAAG-3‘ | 5‘-GAAGAATGGGGCGGAGGAGCTAC-3‘ |
| MOA D2_25 | 5‘-GTGGTGCAGGAACTAGTGCTG-3‘ | 5‘-GTGCGATAAATGATGTTCCTAC-3‘ |
| MOA D2_34 | 5‘-CTTAAATCCCTTACAGATGACC-3‘ | 5‘-GCAACGACGACAACAAGCTAGC-3‘ |
| MOA D2_69 | 5‘-GACGCGCGAGATAATGCTGATG-3‘ | 5‘-CGATGTAGCGACAACCAGGTCC-3‘ |
| MOA D2_75 | 5‘-GAGGGAAACAGAATGTGGAGC-3‘ | 5‘-GACGGCGAATCGCTCCACAGT-3‘ |
| MOA D2_79 | 5‘-GACAATATATGCAGACCTTAAGG-3‘ | 5‘-CATGCTCTGCACCATATGATGC-3‘ |
| MOA D2_83 | 5‘-ACACTTAATAGAAGAGACGTAT-3‘ | 5‘-GAGTCAGTCTCAAGCTACAAAGC-3‘ |
| MOA P C3 | 5‘-TCTGTTTCTTGGTTATACC-3‘ | 5‘-GGTGATTCTTCAGATTCTTA-3‘ |
| MOA P D5 | 5‘-5‘-TCTATATCTTGGCAATAGAA-3‘ | 5‘-CATCCGCCGACTTCTTCCC-3‘ |
| MOA P D2 | 5‘-GTAATGCAAAAGCTGCAATT-3‘ | 5‘-AGTCAAAATATGTAGGGACC-3‘ |
| MOA d0_1 | 5‘-GTGGTGGTGGTAGAGGAAAAG-3‘ | 5‘-GATGAGCCTGACAAAAATTT-3‘ |
| MOA d0_2 | 5‘-GAGCAACCGGCAAATACTCAC-3‘ | 5‘-TGTTCAAAGGGAACTACAAACAC-3‘ |
| MOA d0_23 | 5‘-GTGAAGTGATGAAGACGA-3‘ | 5‘-TGTTCAAAGGGAACTACAAACAC-3‘ |
| MOA d0_30 | 5‘-AATGATCCAAGTGGAAA-3‘ | 5‘-GGAGCAGATGGAACTATA-3‘ |
| MOA d0_37 | 5‘-GCTACACAAATGACGGTGG-3‘ | 5‘-CGAGCAGCTATCAAATTCTA-3‘ |
| MOA D2_33 | 5‘-TAATGAGTTGACGACGAAAA-3‘ | 5‘-ACGTTGTGAGTGGTAATAATT-3‘ |
| MOA D5_2 | 5‘-TGGTGAGTTGACGACGAAAA-3‘ | 5‘-TGACGTGACGTCGACGAGTGGGA-3‘ |
| MOA D5_14 | 5‘-TAGAAAAAAAGTATGGGATGC-3‘ | 5‘-CGACTAATGAAAAATGCCAATG-3‘ |
| MOA D5_77 | 5‘-VGATAAAAAAAAAGCAAAATGGAA-3‘ | 5‘-GGTTGCAGCACAAAAGCACT-3‘ |
| MOA D5_101 | 5‘-CCACAAGAAGGTGCACAAAG-3‘ | 5‘-TCAACGAAGAACGGCGCACA-3‘ |
| MAO C3_2 | 5‘-GGTAGAAATCGGTCTAAAAA-3‘ | 5‘-AGATCAAGTATGGAGAGCTA-3‘ |
| MOA C3_21 | 5‘-ATCACTTGAATCACTTACAGA-3‘ | 5‘-GTAACGCTCAAGGTAATAG-3‘ |
| MOA C3_27 | 5‘-ATTTAGAAAAACGTTTAGAAAC-3‘ | 5‘-CGCGAGATAATGATAAATATTT-3‘ |
| MOA C3_36 | 5‘-ATTGTCAACGAAGAACGGCGC-3‘ | 5‘-CATGTGACGAAGAGAACAAGA-3‘ |
| MOA C3_63 | 5‘-GATAAAGAACGGGAAGAAGC-3‘ | 5‘-CTATTACATGTAAAGCTAAGG-3‘ |
| MOA C3_65 | 5‘-GGCAATTTGAACGGCGCAAA-3‘ | 5‘-AAAGCACTAACATGCGGCGC-3‘ |
| MOA T0_36 | 5‘-CCGAAGAGAAAATTTAGAAAACA-3‘ | 5‘-CCAACATGATGCTCCAGATT-3‘ |
| MOA D7_15 | 5‘-CTTTTCGAGAAATTATATGAAGA-3‘ | 5‘-GCTAATAGAAATGATGTGTGGA-3‘ |
| MOA D7­_33 | 5‘-GAAAAAGACGAATGGGGAGAT-3‘ | 5‘-GGCACTTAATAGACAAGACGT-3‘ |
