# Supplementary material for: In Vitro Variant Surface Antigen Expression in Plasmodium falciparum Parasites from a Semi-Immune Individual Is Not Correlated with Var Gene Transcription
Source: PLoS One. 2016 Dec 1;11(12):e0166135. doi: 10.1371/journal.pone.0166135 (PMC5132323; doi:10.1371/journal.pone.0166135)
Supplement: S3 Table — (DOCX) [file pone.0166135.s008.docx]

| **Parasite line** | **Dominant *var* DBL** | **RCN** |
| --- | --- | --- |
| MOA B5 | d0_37 | 60,11 |
| MOA E8 | d0_37 | 42,61 |
| MOA G3 | d0_37 | 30,8 |
| MOA C4 | C3_36 | 19,54 |
|  | D5_101 | 16,57 |
| MOA E10 | D2_69 | 67,12 |
| MOA J1 | d0_37 | 39,24 |
|  | D2_18 | 13,46 |
| MOA H4 | D7_33 | 37,97 |
|  | d0_37 | 9,08 |
| MOA B10 | D7_33 | 53,92 |
| MOA G2 | T0_36 | 42,7 |
| MOA C8 | T0_36 | 19,88 |
| MOA A1 | T0_36 | 19,31 |
| MOA H6 | T0_36 | 19,25 |
| MOA F11 | T0_36 | 30,93 |
| MOA E1 | C3_65 | 2,82 |
| MOA D11 | D7_15 | 4,95 |
|  | T0_36 | 1,37 |
| MOA G9 | D7_15 | 5,21 |
| MOA bulk | T0_36 | 3,74 |
| MOA D2 | PD2 | 51,81 |
|  | D2_18 | 22,86 |
| MOA D5 | PD5 | 1,17 |
| MOA C3 | D2_75 | 4,26 |
