# Supplementary material for: In Vitro Variant Surface Antigen Expression in Plasmodium falciparum Parasites from a Semi-Immune Individual Is Not Correlated with Var Gene Transcription
Source: PLoS One. 2016 Dec 1;11(12):e0166135. doi: 10.1371/journal.pone.0166135 (PMC5132323; doi:10.1371/journal.pone.0166135)
Supplement: S4 Table — Primer pairs were designed based on the hypervariable regions of the E5 specific DBLs. (DOCX) [file pone.0166135.s009.docx]

S4 Table: **Gene specific q-RT-PCR primer pairs for E5 specific *var* sequences.**

| **primer** | **fwd** | **rev** |
| --- | --- | --- |
| **1 a** | 5'-TCCACGAGCGAGAAGAGGTC-3' | 5'-TCAGAACATGCTGGTCTAAA-3' |
| **5 a** | 5'-CTGCGAATCGAGAAACAGTA-3' | 5'-GACAATATTTACGTCGCCGTTGC-3' |
| **10 a** | 5'-GACGTGACGACAAATGGGAAGA-3' | 5'-AAAATAATGAGCACCCACAT-3' |
| **11 a** | 5'-TGGATTGCATGGGGAAGCAA-3' | 5'-AAAAATAGTTAAAAGCCTTAG-3' |
| **12 a** | 5'-GACAAATTGACACATGATGC-3' | 5'-CCATGTTCGGTGCTTTTTGC-3' |
| **19 a** | 5'-TTAGACAAGAAGTTGAAAGAG-3' | 5'-CCCAGCGCCGCACGTGATGG-3' |
| **23 a** | 5'-GCTAAAAACACGCTACAAAG-3' | 5'-ACCGTGCGCATCACATGTTAATG-3' |
| **29 a** | 5'-TCCTAAAGAGAAAGTACACT-3' | 5'-ATAATAAGCGTTACCTGGTA-3' |
| **35 a** | 5'-GTGGATTGACGAAGAAGA-3' | 5'-TGTTTTTCGAAAATATTGAG-3' |
| **37 a** | 5'-TATATGATAACTTATCTGAT-3' | 5'-ATAATCAGCATCAGTTGGCG-3' |
| **29 a** | 5'-CGAAGGGAGGGAAGAAGTCG-3' | 5'-TATAGTTCCATCTGATCCAT-3' |
| **49 a** | 5'-TTAAATGGAGCTGCAAAATCA-3' | 5'-ATAATTAGCTTTTTGTGGAG-3' |
| **63 a** | 5'-GGAAGTGACGTCTAGCGGGA-3' | 5'-ATGAAAATATGTACCGTGCG-3' |
